# Mechanism of Bacterial Arginine *N*‑Glycosylation: A Chemically Challenging Post-Translational Modification

**DOI:** 10.1021/acscatal.5c07775

**Published:** 2026-01-15

**Authors:** Beatriz Piniello, Ana García-García, Fabio Pietrucci, Ramón Hurtado-Guerrero, Carme Rovira

**Affiliations:** 1 Departament de Química Inorgànica i Orgànica (Secció de Química Orgànica) and Institut de Química Teòrica i Computacional (IQTCUB), Universitat de Barcelona, Martí i Franquès 1, Barcelona 08028, Spain; 2 Institute of Biocomputation and Physics of Complex Systems (BIFI), University of Zaragoza, Mariano Esquillor s/n, Campus Rio Ebro, Edificio I+D, Zaragoza 50018, Spain; 3 Fundación ARAID, Zaragoza 50018, Spain; 4 Copenhagen Center for Glycomics, Department of Cellular and Molecular Medicine, University of Copenhagen, Copenhagen DK-2200, Denmark; 5 Muséum National d’Histoire Naturelle, UMR CNRS 7590, Institut de Minéralogie, de Physique des Matériaux et de Cosmochimie, IMPMC, Sorbonne Université, Paris 75005, France; 6 Institució Catalana de Recerca i Estudis Avançats, Passeig Lluís Companys 23, Barcelona 08010, Spain

**Keywords:** enzymes, *N*-glycosylation, carbohydrates, glycosyltransferases, quantum mechanics/molecular mechanics, metadynamics

## Abstract

Arginine *N*-glycosylation is a post-translational modification that bacterial pathogens use to subvert host immunity, yet the catalytic activation of the intrinsically weak guanidinium nucleophile has remained unresolved. Based on structural data, a direct inverting S_N_2 mechanism had been suggested, but alternative, more stepwise routes and the identity of the catalytic base could not be firmly established. Here, we delineate the molecular mechanism by which the nonlocus of enterocyte effacement (non-LEE)-encoded effector protein B1 (NleB1), a promising virulence factor of enteropathogens, transfers *N*-acetylglucosamine (GlcNAc) to arginine residues of host substrates. Using structural modeling, extensive molecular dynamics, and state-of-the-art QM/MM free-energy simulations combined with kinetic experiments, we elucidate the catalytic mechanism of NleB1. The reaction proceeds through a single-step, dissociative S_N_2-type mechanism, with no stable intermediate. Proton transfer to the catalytic base occurs immediately after the transition state, and is preceded by distortion (loss of planarity) of the acceptor guanidinium that primes nucleophilic attack. The simulations unambiguously identify Glu253, rather than Asp186, as the general base, and reveal that Glu253 plays multiple roles: it disrupts the planar guanidinium conformation of the acceptor arginine to enhance nucleophilicity, orients Arg117, accepts its proton, and subsequently promotes product relaxation via guanidinium replanarization, while Asp186 acts structurally to stabilize the donor substrate. Together, these residues enable a chemically demanding transformation that challenges chemical expectations for guanidinium reactivity. This study provides a comprehensive mechanistic study of arginine *N*-glycosylation, resolving its long-standing mechanistic conundrum and establishing catalytic rules likely conserved among Arg-specific glycosyltransferases.

## Graphical abstract

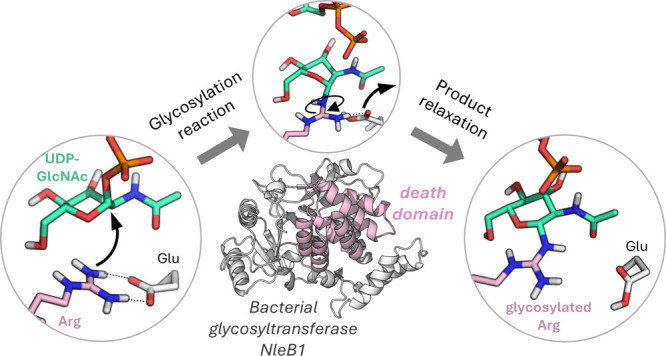

## 1 Introduction

Arginine glycosylation is an unusual post-translational modification that plays a pivotal role in host–pathogen interactions.^1–3^ Several bacterial glycosyltransferases (GTs), including the non-LEE-encoded effector B1 (NleB1) from enterohemorrhagic and enteropathogenic *Escherichia coli* (EHEC and EPEC), its orthologue from *Citrobacter rodentium*, and the *Salmonella enterica* effectors SseK1–3, catalyze the transfer of *N*-acetylglucosamine (GlcNAc) to arginine residues on host proteins.^4–8^ These effector enzymes target a broad range of host proteins, such as glyceraldehyde-3-phosphate dehydrogenase (GAPDH) and death domain-containing proteins including FAS-associated death domain protein (FADD) and TNFR1-associated death domain protein (TRADD). By GlcNAcylating these key regulators, NleB1 inhibits NF-κB signaling, dampening the proinflammatory response and blocking apoptosis, thereby facilitating immune evasion and bacterial persistence during infection (Figure 1).^4–11^ Beyond their host-directed roles, these enzymes also function within the bacterial cell. For example, *C. rodentium* NleB increases bacterial resistance to oxidative stress by GlcNAcylating glutathione synthetase.^9^ Likewise, *S. enterica* effectors SseK1 and SseK3 promote detoxification of methylglyoxal, a toxic metabolic byproduct, by modifying intrabacterial targets.^12^

**Figure 1:**
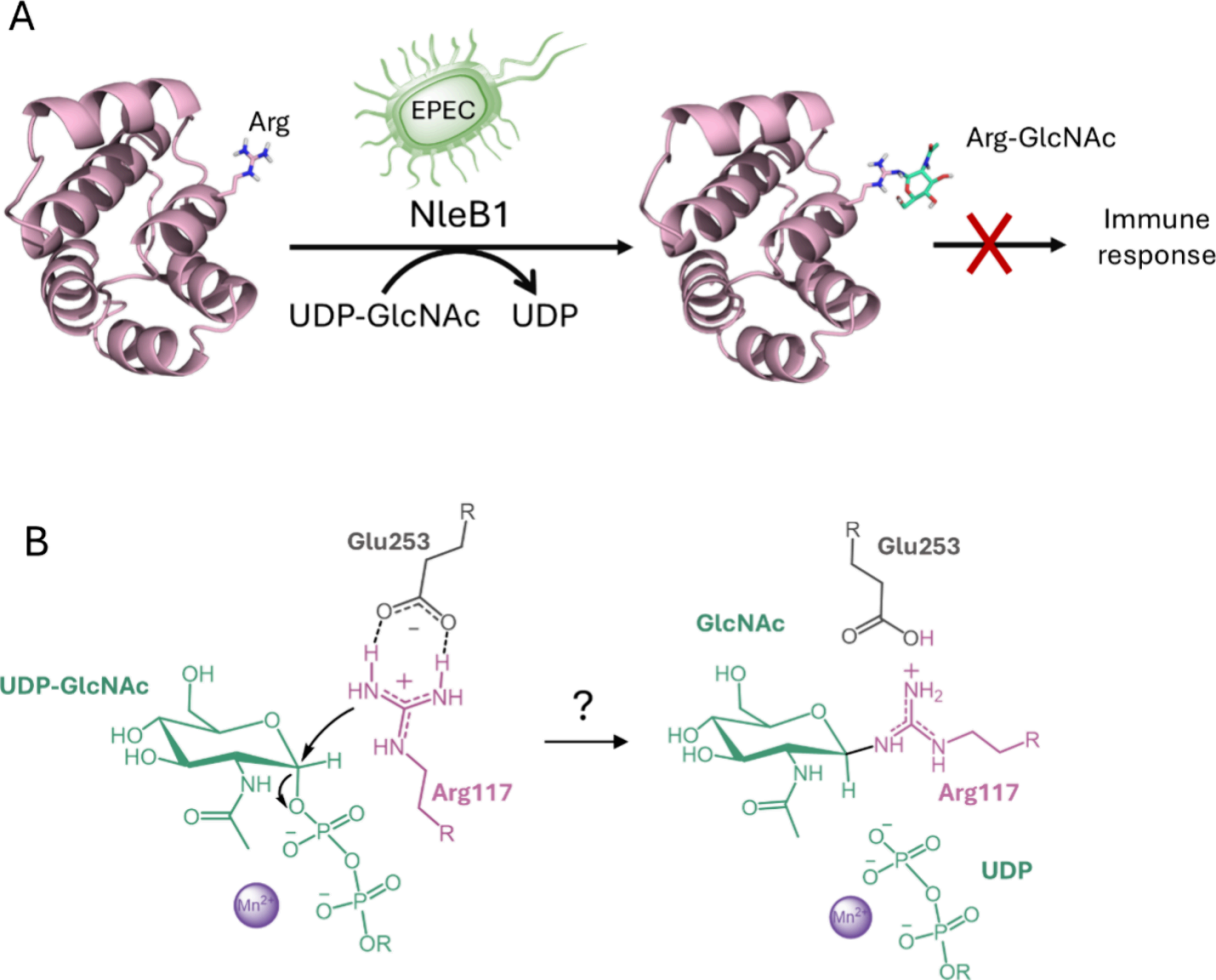
(A) Role of NleB1 in enteropathogenic *E. coli* (EPEC) infection, where it modifies host death domain-containing proteins to block immune signaling. (B) Proposed activation of the acceptor arginine based on the crystal structure of NleB1 in complex with UDP and FADD.^17^ In the putative reactive pose, the guanidinium group is aligned approximately parallel to the mean sugar plane.

Whereas glycosylation typically modifies the side chains of serine or threonine (*O*-glycosylation) or asparagine (“canonical” *N*-glycosylation) residues, NleB1 and its orthologs catalyze the attachment of GlcNAc to arginine residues (Arg *N*-glycosylation). This transformation is chemically challenging because the guanidinium group of the Arg side chain is a poor nucleophile owing to resonance stabilization and its positive charge. Given these properties, arginine glycosylation could, in principle, proceed either through a concerted S_N_2 or a more stepwise S_N_1-like pathway^13^ in which the reaction initiates solely by cleavage of the sugar donor–phosphate bond, forming an oxocarbenium ion intermediate that subsequently collapses with the predeprotonated guanidinium group.

Interestingly, Arg*N*-glycosylation is conserved among effectors from EHEC, EPEC, *C. rodentium*, and *S. enterica*. Notably, EHEC and EPEC also encode a paralog, NleB2, which preferentially transfers glucose rather than GlcNAc,^6,14^ highlighting functional divergence within the family. Crystal structures of NleB1 orthologs SseK1, SseK2 and SseK3 in complex with UDP and/or UDP-GlcNAc,^15,16^ and of NleB1 bound to UDP and FADD,^17^ revealed a typical GT-A fold architecture^15,16^ comprising a central Rossmann-like catalytic core, a protruding helix–loop–helix (HLH) domain, and a C-terminal lid domain. The lid domain exhibits pronounced flexibility -unresolved in the apo structure but ordered in the complexes with UDP or UDP-GlcNAc,^15,16^ indicating that donor binding stabilizes the C-terminal lid and compacts the active site. FADD binding, in turn, occurs only in the presence of the UDP, supporting an ordered Bi–Bi mechanism.^18^ Although the catalytic core is conserved across NleB/SseK, residues mediating acceptor recognition can vary and contribute to differences in substrate selectivity.^18^

In a seminal study, Ding et al. obtained the structure of the pseudo-Michaelis complex of NleB1 with UDP and FADD.^17^ This structure provided a key mechanistic clue: a glutamate residue (Glu253) forms a bidentate interaction with the acceptor arginine (Figure 1A), which may disrupt guanidinium resonance stabilization and enhance nucleophilicity.^17^ Consistent with this model, the Glu253Ala mutation abolishes catalytic activity,^18^ suggesting that Glu253 acts as the catalytic base in an inverting transfer mechanism.^19^ However, the specific substitution reaction -whether the process is truly concerted or involves an oxocarbenium ion intermediate, as might be expected for a weak nucleophile^13^-cannot be inferred from a static structure. Furthermore, another conserved active site residue, Asp186, lies near Arg117 in the crystal structure and is likewise essential for catalysis,^17^ leaving the identity of the true catalytic base ambiguous. Recent studies have further examined the Glu253–Arg117 pairing in the context of substrate promiscuity and ortholog selectivity,^18^ revealing an ortholog-variable second-shell residue, Tyr284 in NleB1 (Ser286/Asn302/Ile289 in SseK1/2/3), that modulates this salt bridge and the acceptor’s orientation. Intriguingly, when suboptimal death-domain–derived peptide substrates are used, NleB-family enzymes can alternatively follow a retaining mechanism^16^ suggesting a substrate-dependent mechanistic plasticity. In the absence of a ternary complex containing both donor and acceptor substrates, however, the precise catalytic mechanism has remained unresolved despite these valuable structural insights.

In this work, building on the available X-ray structures, we constructed the Michaelis complex of NleB1 and elucidated its full catalytic mechanism using a combination of molecular dynamics (MD), hybrid QM/MM simulations (including QM/MM MD, transition path sampling, metadynamics and path collective variable methods) and kinetic measurements. We show that NleB1 operates through a dissociative S_N_2 mechanism in which Glu253 plays multiple catalytic roles: it contributes to disrupt the planar conformation of the guanidinium group of the acceptor arginine to enhance nucleophilicity, acts as a general base during the reaction, and subsequently facilitates product release by enabling restoration of guanidinium planarity and relaxation of the sugar to its most stable ^4^ *C*_1_ chair conformation. Replacement of Glu253 with Asp by site-directed mutagenesis markedly reduces catalytic efficiency, demonstrating that the longer Glu side chain is optimal for positioning, activating, and releasing the arginine acceptor. Together, these findings provide a complete atomistic description of arginine *N*-glycosylation and reveal general enzymatic strategies that enable activation of arginine residues for nucleophilic attack and efficient product turnover.

## 2 Methods

### 2.1 Model Building and Classical MD Simulations

The initial complex was obtained by direct superposition of two PDB structures 6ACI^17^ and 5H63 (Supplementary Figure 1).^16^ The alignment was performed with the STAMP structural alignment algorithm implemented in the MultiSeq plugin of VMD. After system preparation, we performed a classical minimization using the steepest descent method in AMBER18.^20^ The GlcNAc moiety in the PDB structure 5H63 adopts a *cis* conformation of the *N*-acetyl group in one protein subunit and in *trans* conformation in the other. We therefore generated and simulated both possibilities, starting with the one in *cis* and then manually changing it to *trans*. The direct superposition of the two PDB structures (6ACI and 5H63) provided a complete Michaelis complex containing the enzyme, donor (UDP-GlcNAc) and acceptor (FADD). Therefore, no additional structural modeling was required. Minor steric clashes involving the UDP moiety were resolved during the classical minimization. Protonation at pH 7 of residues His, Asp and Glu was determined by MolProbity^21,22^ as well as visual inspection. The topology file was obtained with the AmberTools suite, using force fields FF14SB^23^ (protein, UDP), GLYCAM06 ^24^ (GlcNAc) and TIP3P^25^ (water molecules). The UDP was parametrized (RESP charges)^26^ using antechamber (from AmberTools^27^) and Gaussian09.^28^ A solvation box of dimensions 98.99 × 100.20 × 92.10 Å^3^ was used and 13 Na^+^ ions were added to the system to neutralize the charge. The total number of atoms was 76733, and the number of solvent molecules 23474. Mn^2+^ parameters were taken from the Li et al. ion parameter set for divalent cations using TIP3P water model (12–6 Lennard-Jones parameters).^29^

All classical MD simulations were performed with AMBER18.^20^ The following MD protocol was used. Energy minimization (first of solvent and ions with positional restraints to the protein and ligand, then the whole system) was followed by a stepwise thermalization of the system up to 300 K (100 K of solvent and ions, 100 K of the whole system, followed by increases of 100 K). Afterward, the density was adjusted to 1 g/cm^3^. Finally, the simulation was extended for 500 ns in the NPT ensemble. This procedure (after density adjustment) was repeated three times for each *cis* and *trans* conformers. As we did not observe significant differences in active site structure and dynamics with respect to NAc conformer Supplementary Figures 2 and 3, further simulations and analyses will be discussed only for the most stable *trans* conformer, unless specified. The same procedure was repeated for the Glu253Asp mutant. The same solvation box was used for both WT and mutant enzyme, including 23473 water molecules. Simulations of the product complex were also performed, with three replicas of 250 ns each. The GlcNAc-Arg and UDP were modeled with ff14SB and GAFF2. The same simulation procedure as for the ternary complexes simulations was used. The dimensions of simulation box were 93.50 × 94.64 × 86.992 Å^3^, including 23474 water molecules. The time evolution of the root-mean-square deviation (RMSD) during the production MD simulations are provided in Supplementary Figure 2.

### 2.2 QM/MM MD Simulations

The QM/MM starting snapshot was selected based on representative average distances between key residues and substrates obtained from MD simulations. While additional starting configurations could have been considered, we do not expect substantial differences in the reaction mechanism. The CP2K code,^30^ patched with PLUMED 2.8,^31^ was employed. In CP2K, the QM/MM calculations are performed with a combination of QUICKSTEP^32^ (QM) and FIST^30^ (MM) codes. We used the PBE functional^33^ with DFT-D3 ^34^ corrections. This functional was chosen as it provides a reasonable balance between accuracy and computational cost. It is also consistent to previous studies of sugar puckering, an important issue in cabrohydrate-active enzymes.^35^ This is also the functional that we and others have successfully used in earlier work on carbohydrate-active enzymes.^36–38^

The QM region (Supplementary Figure 4) comprised the GlcNAc molecule, part of UDP, Mn^2+^ and its coordination sphere, and the side chains of Arg117 and Glu253 (90 atoms in total). The simulations were performed in the sextet spin state, as corresponds to a high-spin Mn^2+^ configuration. The QM region was defined according to the following criteria: (i) all atoms directly involved in the reaction were included in QM, together with Mn^2+^ and its complete coordination sphere (side chains of Asp209, Asn306, and Ser308, the phosphate groups, and the coordinating water molecule); (ii) the QM–MM boundaries were chosen as C­(sp3)–C­(sp3) to minimize boundary polarization effects; and (iii) the QM box size was optimized to achieve a balance between completeness and computational cost. Classical MD simulations excluded the direct participation of other residues, most notably Asp186, in catalysis, and its inclusion in the QM region was therefore deemed unnecessary.

The boundary between QM and MM partitions was modeled via link H atoms, using the Integrated Molecular Orbital Molecular Mechanics (IMOMM) method.^39^ The QM box dimensions were taken as 16.76 × 23.61 × 18.49 Å^3^. The Gaussian and plane-waves (GPW) scheme was used, with a plane-wave cutoff of 350 Ry and relative cutoff of 60 Ry, the Gaussian triple-ζ valence polarized (TZV2P) basis set, and GTH pseudopotentials.^40^ The simulations were performed in the NVT ensemble, using a Nosé-Hoover thermostat^41^ at 300 K and a time step of 0.5 fs. Nonbonded interactions cutoff was taken as 12 Å. The simulation protocol consisted in geometry optimization (conjugate gradients for 1500 steps and LBFGS (1147 steps), followed by heating of the system up to 300 K and QM/MM MD equilibration (10 ps). The RMSD time evolution of the atoms in the QM region, as well as the evolution of selected distances can be found in Supplementary Figure 16.

### 2.3 Initial Metadynamics Simulation with One CV

To activate the reaction and model the transition from reactants to products, we used metadynamics^42,43^ coupled to QM/MM MD.^44^ As a first test, we used one collective variable (CV) , taken as the difference between the attacking distance (N_η2_–C1) and the glycosidic bond breaking distance (C1–O_P_), to drive the reaction. The Gaussian height was taken as 1 kcal/mol, the width as 0.1 Å and the deposition pace as 80 MD steps (40 fs). We were able to obtain the product state, in which a new bond between the Arg and C1 of GlcNAc was formed and one of the protons linked to the attacking N_η2_ atom was abstracted by Glu253. The simulation was stopped after one recrossing over the TS (Supplementary Figure 5).

### 2.4 Two-Node Path CV Metadynamics Simulation

The reaction coordinate was further assessed using path CV metadynamics.^45,46^ In this method, several geometric descriptors are combined into a single CV with two components: *s*, representing the progress along the path, and *z*, representing the deviation from it. The path is discretized into several states (nodes) that define the putative reaction coordinate. As a first approximation, we used only two nodes – reactant and product states obtained in our previous 1-CV metadynamics–, following the approach of Branduardi et al.^46^ Geometrical descriptors were defined as combinations of coordination numbers corresponding to key bond-forming and bond-breaking events during the **MC** → **P** transition, following the method of Pietrucci and Saitta^47^ (see Supplementary Tables 1A and 1B). The following formula was taken for the coordination numbers:

$${\mathrm{CN}}_{\mathrm{ij}}=\frac{1-{\left(\frac{r_{\mathrm{ij}}}{r_{0}}\right)}^{n}}{1-{\left(\frac{r_{\mathrm{ij}}}{r_{0}}\right)}^{m}}$$ 1

where CN_ij_ is the coordination number between atoms i and j, and r is the interatomic distance. The parameters *n* and *m* were set to 6 and 12, respectively; *r*_0_ was taken as 2.1 for bonds not involving hydrogen, and 1.2 otherwise. The metadynamics parameters were taken as follows: Gaussian height of 2 kcal/mol, Gaussian width of 0.05 (on *z*) and 0.1 Å (on *s*). A deposition pace of 50 MD steps (25 fs) was considered. The lambda parameter was fixed at 0.48, and a wall potential was applied on the *z* component at 0.7. The resulting reaction mechanism closely matched that previously obtained from the 1-CV metadynamics. However, the simulation did not converge, as no recrossing over the TS was observed. We therefore increased the number of nodes used in the path CV from 2 to 20. Nevertheless, we used the TS identified in the 2-node simulation as the starting point for exploration of the TS ensemble. The metadynamics free energy landscape obtained from the 2-node path CV simulation can be found in Supplementary Figure 17.

### 2.5 Analysis of the TS Ensemble Using Aimless Shooting Simulations

The TS ensemble was explored using aimless shooting.^48,49^ In this technique, we start from a trajectory that successfully connects reactants and products, which in our case was obtained from the 2-node path CV simulation. From this trajectory, we selected several configurations near the TS (specifically, a small number of MD steps away, both before and after the TS). For each configuration, we launched (“shoot”) two new unbiased QM/MM MD simulations with equal velocity (sampled from a Maxwell–Boltzmann distribution) but opposite directions. Trajectories that evolve toward the same state (either reactants or products) are rejected, while those reaching both states are accepted, selecting the new configurations from the newly accepted trajectory. In this way, we iteratively explore the transition state ensemble. A total of 200 shootings were launched. The shooting time step, which defines the subsequent shooting configuration, was taken as 10 fs (20 MD steps) at first, and later increased to 15 fs (30 MD steps) because the initial choice resulted in an acceptance ratio too high. For the first case, 50 shootings were launched (33 accepted trajectories; 66%). For the second one, we launched 150 shootings (62 accepted trajectories; 41%). The resulting TS ensemble was analyzed in terms of the most relevant distances that define the catalytic reaction Supplementary Figure 6.

### 2.6 Multiple Nodes Path CV Metadynamics Simulations

The trajectories obtained from aimless shooting were processed into a new path expanding 20 nodes. The path metadynamics method developed by Díaz Leines and Ensing,^45^ which enables on-the-fly modification of the path, was employed. The simulation was run for 24 ps, during which 640 Gaussians were deposited. The following metadynamics parameters were used: Gaussian width of 0.02 Å, deposition stride of 75 MD steps (37.5 fs), and Gaussian height of 0.6 kcal/mol. The path was updated every 750 MD steps (375 fs). The evolution of the CV and the path during the simulation is shown in Supplementary Figure 9.

The simulation was stopped upon the first recrossing over the TS, from products back to reactants, as recommended for chemical reactions.^50^ In our experience, this minimizes the risk of exploring undesired side reactions that would lead to simulation hysteresis. Although this protocol does not allow precise quantification of the statistical error on the free energy barrier, it provides reliable mechanistic insight. Notably, recent work on a glycosidase, for which multiple recrossings could be achieved, showed that the standard error is relatively modest (within ± 1 kcal/mol) and that the mechanism remains unaltered.^51^ The reaction mechanism obtained from the optimized path was consistent with the dissociative S_N_2 reaction previously observed in the 1-CV metadynamics simulation, indicating robustness of the mechanistic outcome with respect to the sampling method. The computed energy barrier was in good agreement with the experimental value (19 vs 17 kcal/mol, respectively).

Additional committor analyses were performed to validate the transition state configuration obtained in the (20-nodes) path CV metadynamics simulation Supplementary Figure 9. Ten different configurations in a small region around the putative TS were selected and ten unbiased MD runs were performed for each configuration. The best TS configuration gave a 60/40 probability of falling into reactants/products. The TS was further refined by aimless shooting simulations Supplementary Figure 10. The main distances defining the active state structure in the MC, TS and P states can be found in Supplementary Table 2.

### 2.7 Relaxation of the Reaction Products

The relaxation of the product was investigated with QM/MM metadynamics. The CVs were taken as the distance between the attacking N_η2_ atom of the Arg117 side chain and the gamma carbon of Glu253 (CV1), as well as the torsion between atoms C1–N_η2_-C_ζ_-N_ε_ (CV2). The metadynamics parameters were the following: Gaussian width of 0.1 (CV1) and 0.07 Å (CV2), Gaussian height of 0.4–0.8 kcal/mol, and deposition time of 80 MD steps (40 fs). A total number of 2970 Gaussians were deposited (118 ps). The metadynamics simulation started from a configuration of the products state obtained from the previous 20-node path CV metadynamics simulation, after 4 ps of unbiased QM/MM MD. A parabolic potential wall with a force constant of 150 kcal/mol was applied to CV1 at 6.5 Å, in order to avoid the sampling of less relevant configurations. The simulation was stopped after recrossing of both CVs back to the reactants state ( Supplementary Figure 13).

### 2.8 Protein Expression and Purification

The DNA sequences encoding the amino acid residues of NleB1 from enterohemorrhagic *E. coli* (EHEC) were codon-optimized and synthesized by GenScript (USA) for expression in *E. coli* . The DNA constructs include a *Pst*I recognition site at the 5′ end and a *BstE*II recognition site with a stop codon at the 3′ end. NleB1 EHEC was inserted into the pMALC2x vector as a fusion protein, resulting in the vector pMALC2x-12His-TEV-*NleB1­(EHEC)*, which includes a Tobacco Etch Virus (TEV) protease cleavage site between the maltose-binding protein (MBP) and NleB1. Site-directed mutagenesis performed by GenScript was used to generate the NleB1 EHEC E253D mutant using the same vector.

Each plasmid was transformed into *E. coli* BL21­(DE3) cells and cultured in 2xTY medium (1.6% tryptone, 1% yeast extract, 0.5% NaCl, w/v) supplemented with 100 μg/mL ampicillin at 37 °C. Induction was initiated with 1 mM IPTG when optical density at 600 nm (OD600) reached 0.6–0.8, and cultures were incubated at 18 °C for 16 h. Cells were then harvested by centrifugation at 10,000 rpm for 10 min at 4 °C, lysed in Buffer A (25 mM Tris pH 7.5, 500 mM NaCl, 10 mM imidazole), and the lysate was loaded onto a His-Trap column (GE Healthcare). Proteins were eluted with a gradient of imidazole from 10 mM to 500 mM and dialyzed into Buffer C (25 mM Tris pH 7.5, 150 mM NaCl). TEV protease was then used to cleave at the TEV site, and proteins were concentrated using an Amicon Ultra-15 mL centrifugal filter. Protein concentration was determined by UV absorbance at 280 nm, using a theoretical extinction coefficient of 55810 M^–1^ cm^–1^.

### 2.9 Kinetic Analysis

Kinetic parameters for wild-type NleB1 and the mutant Glu253Asp were assessed using the UDP-Glo luminescence assay (Promega). Assay conditions included 10 nM NleB1 or E253D mutant, 25 mM Tris pH 7.5, 150 mM NaCl, 50 μM MnCl_2_, and 500 μM UDP-GlcNAc with varying concentrations of FADD from 5 to 1000 μM. Reactions were incubated at 37 °C for 20 min and stopped by adding 5 μL of UDP detection reagent. The mixtures were then incubated in a white 384-well plate in the dark at room temperature for 1 h before luminescence measurement on a Synergy HT microplate reader (Biotek). To estimate the amount of UDP produced in the glycosyltransferase reaction, we created a UDP standard curve. The values were corrected against the UDP-GlcNAc hydrolysis and were fit to a nonlinear Michaelis–Menten with substrate inhibition program in GraphPad Prism 6 software from which the *K*_*m*_, *k*_*cat*_, *V*_*max*_ and *K*_*i*_ values along with their standard deviations were obtained. All experiments were performed in duplicate following Promega’s UDP-Glo recommendations and due to the limited amount of FADD available. The wild type and the Glu253Asp mutant both exhibited a distinct substrate inhibition profile when exposed to varying concentrations of FADD, showing remarkably similar *K*_*i*_s (Figure 5; *K*_*i*_ = 1291 ± 273.4 vs 468.3 ± 358.2 μM, respectively). Raw experimental velocity and substrate concentration data, along with corresponding residuals from kinetic model fitting, are provided in Supplementary Table 3.

## 3 Results

### 3.1 Structure and Dynamics of the Michaelis Complex

A structure of NleB1 containing all components required for catalysis — the donor substrate (UDP-GlcNAc), the acceptor substrate (FADD domain) and the Mn^2+^ cofactor—is not available. We therefore constructed the Michaelis complex by structural overlay of two high resolution complexes: NleB1 bound to UDP and the FADD death domain (PDB 6ACI),^17^ in which the sugar donor is missing; and the NleB ortholog SseK2 bound to UDP-GlcNAc (PDB 5H63),^16^ in which the acceptor is missing. The Mn^2+^ cation was present in both structures. The enzyme-FADD coordinates were taken from the NleB1-UDP-FADD complex, and the UDP-GlcNAc-Mn^2+^coordinates from the SseK2-UDP-GlcNAc-Mn^2+^ complex Supplementary Figure 1.

The resulting Michaelis complex (Figure 2 and Supplementary Figure 1) positions Arg117 for in-line attack on the anomeric carbon of the donor GlcNAc. The C1–N_η2_ distance is 4.5 Å and the N_η2_–C1-O_P_ angle is 177°, close to the ideal 180° for an S_N_2 reaction. Glu253 is close to the guanidinium group of Arg117, suggesting a catalytic role.^17^ A second acidic residue, Asp186, lies near Arg117 (at 4.2 Å from oxygen to hydrogen) and could potentially interact with it during the enzyme dynamics. Therefore, the static structural overlay alone does not exclude the participation of either Glu253 or Asp186 as a catalytic base.

**Figure 2:**
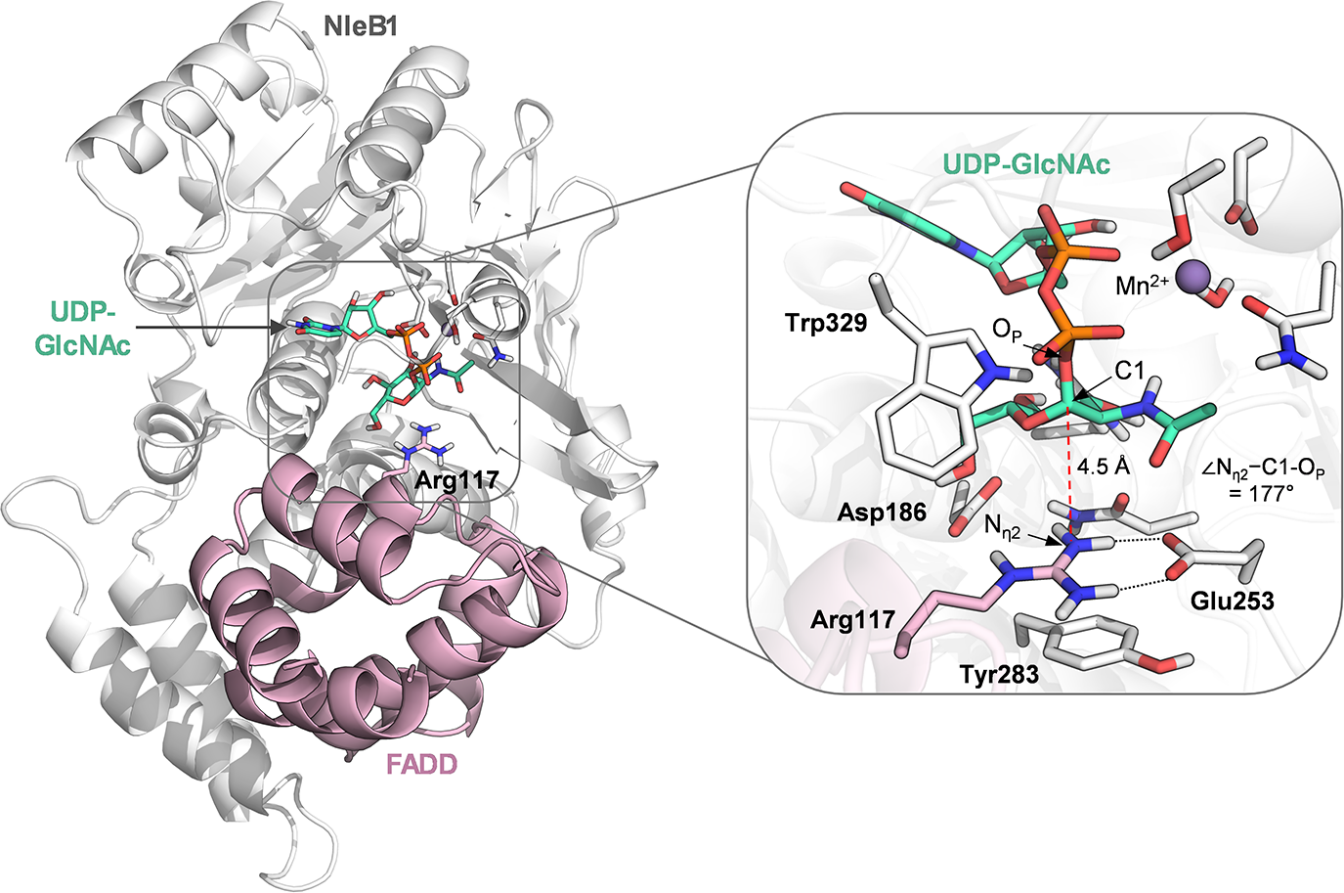
NleB1 in complex with FADD and UDP-GlcNAc-Mn^2+^ obtained from structural overlay of two crystal structures: NleB1 in complex with UDP and FADD (PDB entry 6ACI)^17^ and SseK2 in complex with UDP-GlcNac (PDB entry 5H63).^16^

Classical MD simulations with AMBER18 ^20^ (three 500 ns replicas; Supplementary Figure 2) showed that the ternary complex is stable during the simulation time scale. Arg117–Glu253 forms a bidentate interaction, with occasional flips of the Glu253 oxygen atoms (Supplementary Figure 3). Asp186 moved during the dynamics to interact with the C4 and C6 hydroxyl groups of GlcNAc, preventing Arg117 from contacting both acidic residues simultaneously. This makes Asp186 to be poorly positioned for proton abstraction from Arg117, making its role as a general base unlikely. Notably, mutational data indicate that Asp186 is nevertheless essential for enzymatic activity: the Asp186Ala variant is catalytically inactive.^17^ This suggests that Asp186 has a structural or substrate stabilizing role rather than direct chemical participation in catalysis. Overall, MD simulations indicate that the catalytically competent configuration involves Arg117 interacting exclusively with Glu253. In this configuration, the C1–N_η2_ distance averages 4.3 Å (Supplementary Figure 3), and the N_η2_–C1–O_P_ angle remains close to 160°, an arrangement favorable for nucleophilic substitution.

### 3.2 Reaction Mechanism

We selected a representative frame of the most frequently sampled configuration in the classical MD simulations and performed QM/MM MD equilibration (see Methods). The QM region (Supplementary Figure 4) comprised the GlcNAc and phosphate atoms of UDP, the Mn^2+^ ion with its full coordinating sphere, the side chain of the acceptor Arg117, and the side chain of Glu253 (90 QM atoms). The resulting structure is shown in Supporting Figure 4.

To explore the reaction pathway, we first applied QM/MM metadynamics^42^ using a single collective variable (CV) defined as the difference between the distance of nucleophilic attack (C1–N_η2_) and the distance for glycosidic bond cleavage in UDP-GlcNAc (C1–O_P_). This CV does not include any coordinate related to arginine deprotonation; thus, the simulation did not preassign the identity of the general base. This choice allowed us to observe, without bias, which residue would most likely accept the proton during the reaction. The free energy profile (Supplementary Figure 5) supports a concerted S_N_2 reaction. Notably, there is no intermediate basin or plateau along the reaction coordinate, ruling out a stepwise S_N_1-type mechanism. In this concerted S_N_2 reaction, a new C1–N_η2_ bond formed and one proton from the attacking nitrogen atom was transferred to Glu253 after the transition state (TS), revealing its role as the general base (Supplementary Figure 5). However, the calculated free energy barrier (22 kcal/mol) was higher than the experimental estimate (17 kcal/mol),^18^ likely due to the suboptimal CV choice, prompting further refinement of the reaction coordinate.

We next employed path-metadynamics,^45^ which combines multiple geometric descriptors into a single CV with two components: *s* (reaction progress) and *z* (deviation from the path). The descriptors, defined as coordination numbers between specific atom pairs, as suggested for chemical reactions,^47^ capture the key chemical events: (i) cleavage of the C1–O_P_ glycosidic bond of UDP–GlcNAc, (ii) formation of the C1–N_η2_ bond between the donor sugar and the acceptor arginine, (iii) hydrogen bond interactions between Arg117 and Glu253 (O1/O2 interacting with H_η1_ and O1/O2 interacting with H_η2_), and (iv) transfer of a proton from the attacking nitrogen (N_η2_) to the catalytic base. Together, these descriptors ensure that the path CV simultaneously tracks all bond breaking and bond formation events along the Michaelis complex (**MC**) → product (**P**) transformation. Using the outcome of the previous 1-CV metadynamics simulation as initial guess for the path, we performed an initial path CV QM/MM metadynamics run. The path was then improved through aimless shooting simulations (see Supplementary Figure 6 and Methods), followed by a final path CV metadynamics simulation. The evolution of the path CV, as well as the results of aimless shooting simulations starting from the TS can be found in Supplementary Figures 7–9.

The free energy profile of the glycosylation reaction (Figure 3A) is again indicative of a single step reaction, i.e. a concerted reaction (S_N_2) leading to inversion of configuration. The computed free energy barrier (19 kcal/mol) agrees with the value estimated from previous experimental rates^18^ and from this work (17.4 kcal/mol at 37 °C; see Methods).

**Figure 3:**
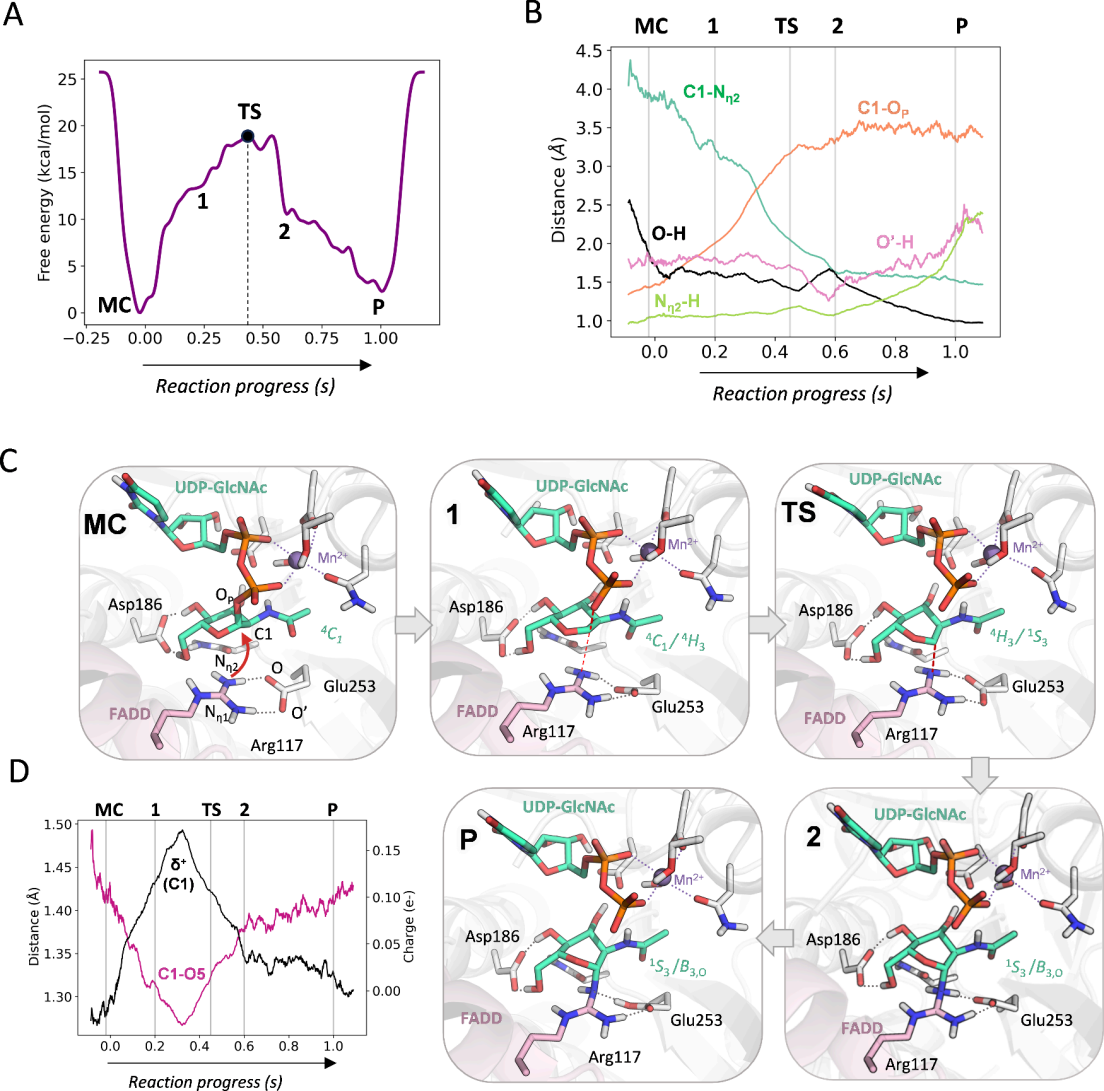
(A) Free energy profile of the glycosyltransfer reaction catalyzed by NleB1. (B) Distances along the reaction coordinate, corresponding to the *s* component of the path CV. (C) Representative snapshots of the main states along the reaction coordinate. Dashed black lines indicate bonds that are being broken or formed, whereas gray dotted lines indicate hydrogen bonds or coordination bonds involving Mn^2+^. (D) Evolution of the anomeric charge and the C1–O5 bond distance of the GlcNAc donor along the reaction coordinate (O5 being the sugar ring oxygen). A running average window of 20 frames was applied for clarity.

Representative snapshots along the reaction coordinate (Figure 3C) show that the first major event is UDP–C1 bond cleavage, preceding formation of the new C1-Arg bond. In the MC state, the C1–N_η2_ distance is ∼ 4 Å. At state **1**, where the free energy profile changes curvature, the C1–O_P_ bond increases to ∼ 2 Å (Figure 3B) and the sugar ring distorts from its stable ^4^ *C*_1_ chair to a ^4^ *H*_3_ half-chair conformation. Although the C1–N_η2_ distance remains relatively long (∼3 Å), the arginine N_η2_ begins to interact with the anomeric carbon, as evidenced by the pyramidalization of the NH_2_ group, leading to guanidinium distortion (Supplementary Figure 10). The transition state (*s* ≈ 0.45) features a fully broken UDP–C1 bond (3.40 Å) and a partially formed C1–N_η2_ bond (2.04 Å; Supplementary Table 2), indicating that the TS is highly dissociative,^52,53^ in line with other inverting glycosyltransferases.^36,38^ The GlcNAc is in a ^4^ *H*_3_/^1^ *S*_3_ conformation and the C1–O5 bond is shorter compared to the MC state, indicating the formation of an oxocarbenium ion-like species (Figure 3D).

Formation of the *N*-glycosidic bond becomes complete at state **2** (C1–N_η2_ ≈ 1.6 Å). Subsequently, the H_η2_ proton oscillates between Arg117 and Glu253, including transient transfer of the other H involved in the bidentate interaction (H_η1_) to Glu253. This hydrogen bond dynamics underscores the strong Arg–Glu interaction. The reaction ends with H_η2_ transfer to Glu253, yielding the glycosylated arginine product (see Supplementary video 1). Overall, these simulations show that NleB1 catalyzes arginine glycosylation via a dissociative S_N_2 reaction mechanism. Glu253 functions as a general base, with proton transfer occurring after the TS. Critically, guanidinium distortion precedes the TS, aided by the strong bidentate Arg117–Glu253 interaction that primes arginine for nucleophilic attack.

### 3.3 Product Relaxation

As shown in the free energy profile of Figure 3A, the energy difference between the MC state (**MC**) and the reaction products (**P**) is positive (2.3 kcal/mol), indicating an apparently unfavorable reaction. However, analysis of the structure of **P** suggests that it may not correspond to the final product state. First, the GlcNAc sugar donor remains in a distorted ^1^ *S*_3_/*B*_3,O_ conformation (Figure 3C) rather than its most stable ^4^ *C*_1_ conformation in solution. Second, the guanidinium group of the glycosylated arginine is significantly nonplanar, lacking full resonance stabilization. These features suggest a subsequent relaxation step in which guanidinium planarity is restored and the sugar changes to the ^4^ *C*_1_ conformation. However, it is unclear whether this occurs during product unbinding or after the product is released from the active site into solution.

To investigate the relaxation of the reaction product, we performed MD simulations (three replicas of 250 ns) starting from a snapshot of the **P** state (Figure 3A). Within the first 10 ns, the guanidinium group recovered the planarity and the GlcNAc moiety evolved to the ^4^ *C*_1_ chair conformation. Remarkably, Glu253 detached from Arg117 during this process. The Arg117–Glu253 distance increased by ∼ 2 Å, coinciding with both guanidinium planarization and sugar relaxation (Supplementary Figure 11). This indicates that product relaxation is driven by the disruption of the Arg–Glu253 interaction, and does not require major rearrangements in the C-terminal region of the enzyme, despite this region being disordered in the structure of the unliganded enzyme.^17^

Further insight was obtained from QM/MM metadynamics using two CVs: (i) the Arg117–Glu253 distance, and (ii) a dihedral angle describing the planarity of the arginine guanidinium side chain (Figure 4). The resulting free energy surface revealed three distinct regions, each containing several local minima. State **1** corresponds to the unrelaxed **P** state, with distorted guanidinium (Ω ≈ 110°) and Glu253 tightly bound to Arg117. In state **2**, the guanidinium is still distorted but Glu253 has partially separated from it (d = 5.8 Å). In state **3** (the global minimum, ≈ 12 kcal/mol lower in energy than state **1**), Glu253 is fully detached (d = 6.5 Å) and Arg117 has regained guanidinium planarity. Furthermore, the sugar ring has also recovered the chair conformation (Figure 4B and Supplementary Figure 12). The lower energy of state **3** compared to the **MC** state makes the overall glycosylation reaction exergonic by ≈10.5 kcal/mol. These results reveal that Glu253 not only serves as the catalytic base and “orientator” of the guanidinium nucleophile, but also facilitates product release. Glu253 detachment is the driving force for product relaxation, enabling both restoration of guanidinium planarity and relaxation of the sugar to the ^4^ *C*_1_ conformation (see also Supplementary video 2). These results show that late-stage rearrangements, beyond the chemical step itself, play a pivotal role in NleB1 turnover.

**Figure 4:**
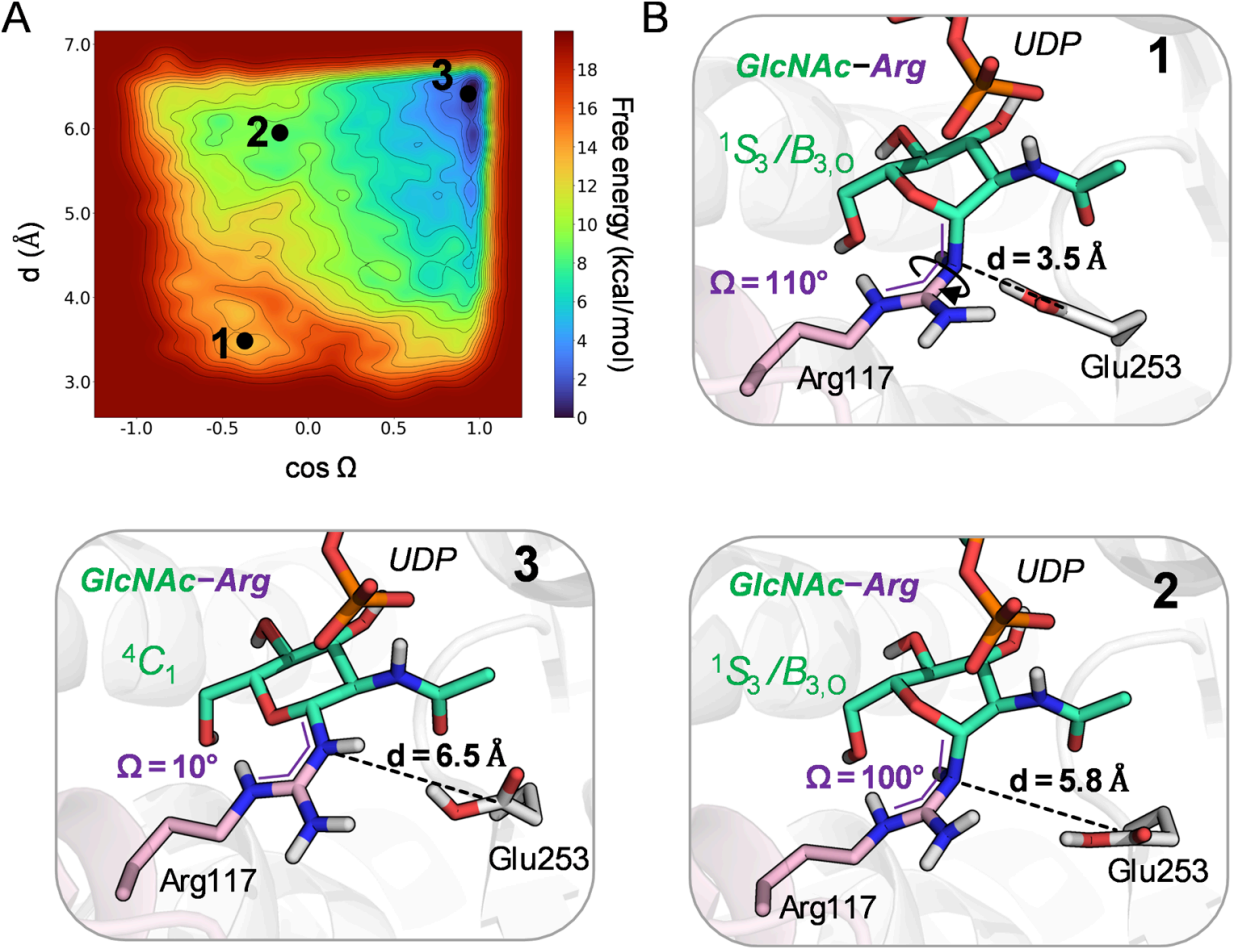
(A) Free energy landscape (FEL) corresponding to the relaxation of the reaction product. Isolines at 2 kcal/mol. (B) Snapshots along the FEL, highlighting the sequence of events: separation of Glu253 from Arg117 and rotation of the arginine guanidinium group. The evolution of the two CVs during the simulation can be found in Supporting Figure 13.

### 3.4 Site-Directed Mutagenesis Confirms the Critical Role of Glu253

The role of Glu253 in catalysis was further investigated by substituting this residue with aspartate, a conservative change that would be expected not to greatly alter the active site architecture. However, this turned out not to be the case. The Glu253Asp mutation increased *K*_*m*_ by ≈ 20-fold (244 ± 155 μM vs 12.25 ± 2.2 μM) and reduced enzymatic activity by ≈ 6.5-fold compared to the wild type (*k*_*cat*_ of 19.35 ± 8.6 vs 127.28 ± 7.5 min^–1^; Figure 5A), resulting in an overall ≈ 131-fold decrease in catalytic efficiency (*k*_*cat*_/*K*_*m*_). Therefore, although the Glu253Asp mutant retains some activity, it is significantly less efficient in both catalysis and substrate binding.

**Figure 5:**
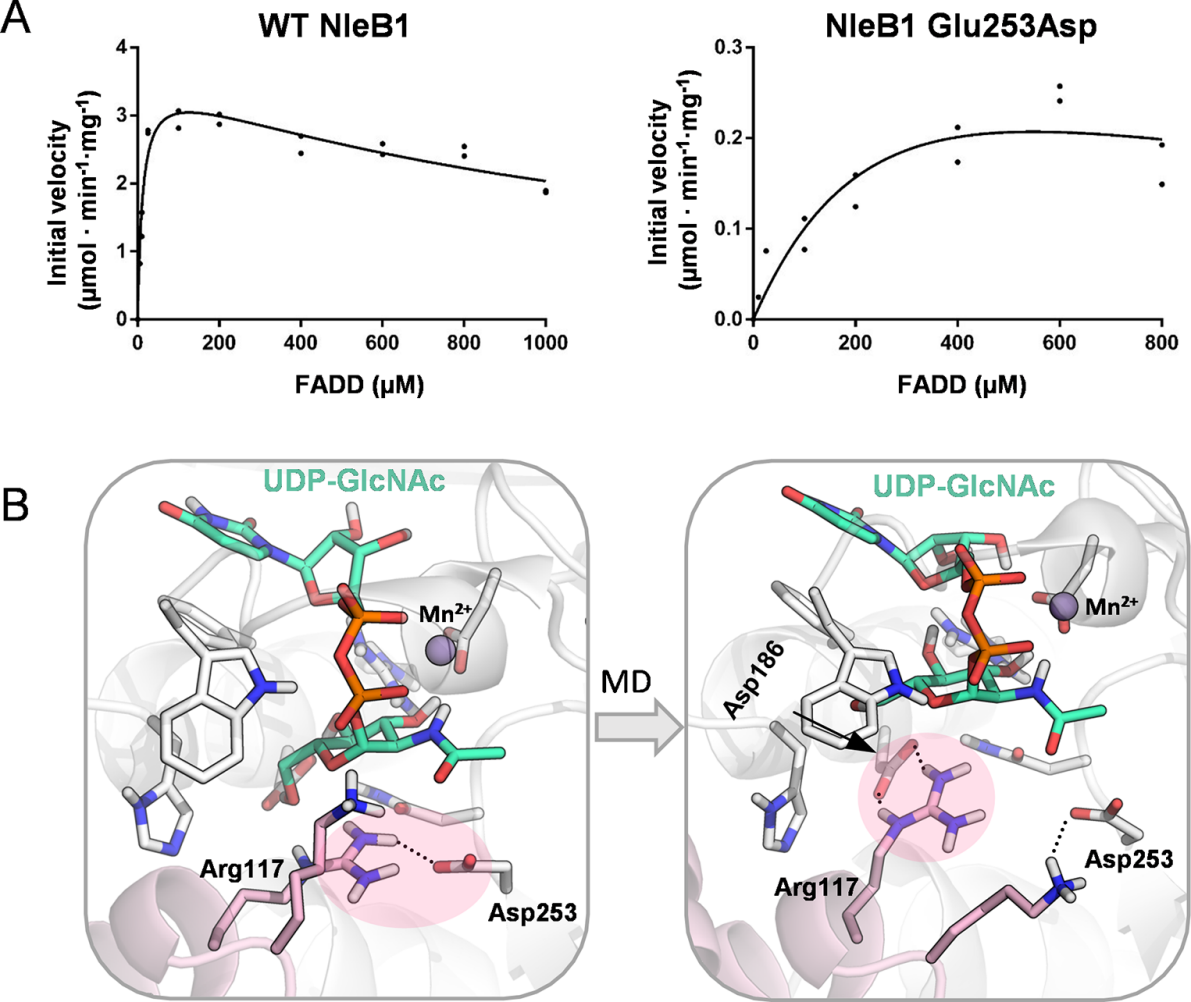
(A) Kinetic assays for wild-type NleB1 and the Glu253Asp mutant at 37 °C, showing reduced catalytic turnover and substrate affinity in the mutant. (B) Representative MD snapshots of the Glu253Asp mutant, illustrating loss of the Arg117–Glu253 bidentate interaction and suboptimal geometry for nucleophilic attack.

To investigate the effect of the mutation on the active site structure and dynamics, we performed MD simulations (three replicas of 500 ns) of Glu253Asp NleB1 in complex with the donor and acceptor substrates. In the mutant, the Arg117–Asp253 interaction proved much less stable than the Arg117–Glu253 salt bridge in the wild-type enzyme (Supplementary Figure 14). Specifically, the N_η2_–C1 distance elongates (Supplementary Figure 14) and the guanidinium group adopts an almost perpendicular orientation relative to the donor sugar (Figure 5B) in contrast with the wild type enzyme. We also observed that Asp186 moves to interact tightly with Arg117. However, this positions the guanidinium group in an unfavorable orientation for nucleophilic attack (Figure 5B). This misalignment likely weakens the Asp186–GlcNAc interaction that was observed in the WT enzyme and perturbs the active-site geometry, thereby providing a plausible basis for the elevated *K*_m_ of Glu253Asp.

These results confirm that the native Glu side chain is not only optimal for catalysis, but also for enabling productive product release. In the wild type, the Glu side chain supports optimal positioning of Arg117 during catalysis and later detaches to allow guanidinium planarization and sugar relaxation. The shorter Asp side chain compromises both the catalytic and product release steps. Other differences between Asp and Glu beyond side chain length may also contribute to catalysis, such as the slightly higher p*K*_a_ of Glu that makes it more likely to accept a proton during the reaction. Altogether, this explains the marked loss of catalytic efficiency observed experimentally for Glu253Asp NleB1.

## 4 Discussion

Bacterial glycosyltransferase non-LEE encoded effector protein B1 (also known as NleB1) catalyzes the *N*-glycosylation of arginine residues in proteins, promoting infection by disrupting the host immune response. A central mechanistic question is how the enzyme enables arginine —an intrinsically poor nucleophile due to the resonance-stabilized guanidinium group— to react with the donor sugar. Here, we have investigated this reaction in detail, combining computational and experimental approaches.

Our results show that Glu253 plays multiple roles across the catalytic cycle: it activates the nucleophile and later promotes product release. In the Michaelis complex, Glu253 serves as a general base and interacts bidentately with both N_η_ atoms of Arg117. This interaction not only activates the nucleophile but also maintains the optimal geometry of the Michaelis complex – with the guanidinium group parallel to the sugar plane (see Figures 3C and 6)- contributing to the disruption of guanidinium planarity and its resonance stabilization during the reaction, thereby priming Arg117 for nucleophilic attack on the partially positively charged anomeric carbon (Figure 3C). In the products complex, once the reaction has taken place and Arg117 becomes glycosylated, Glu253 facilitates product release by detaching from Arg117. This allows restoration of guanidinium planarity and relaxation of the sugar to its stable ^4^ *C*_1_ chair conformation. The proposed reaction scheme, highlighting the key electronic and structural rearrangements, is shown in Figure 6.

**Figure 6:**
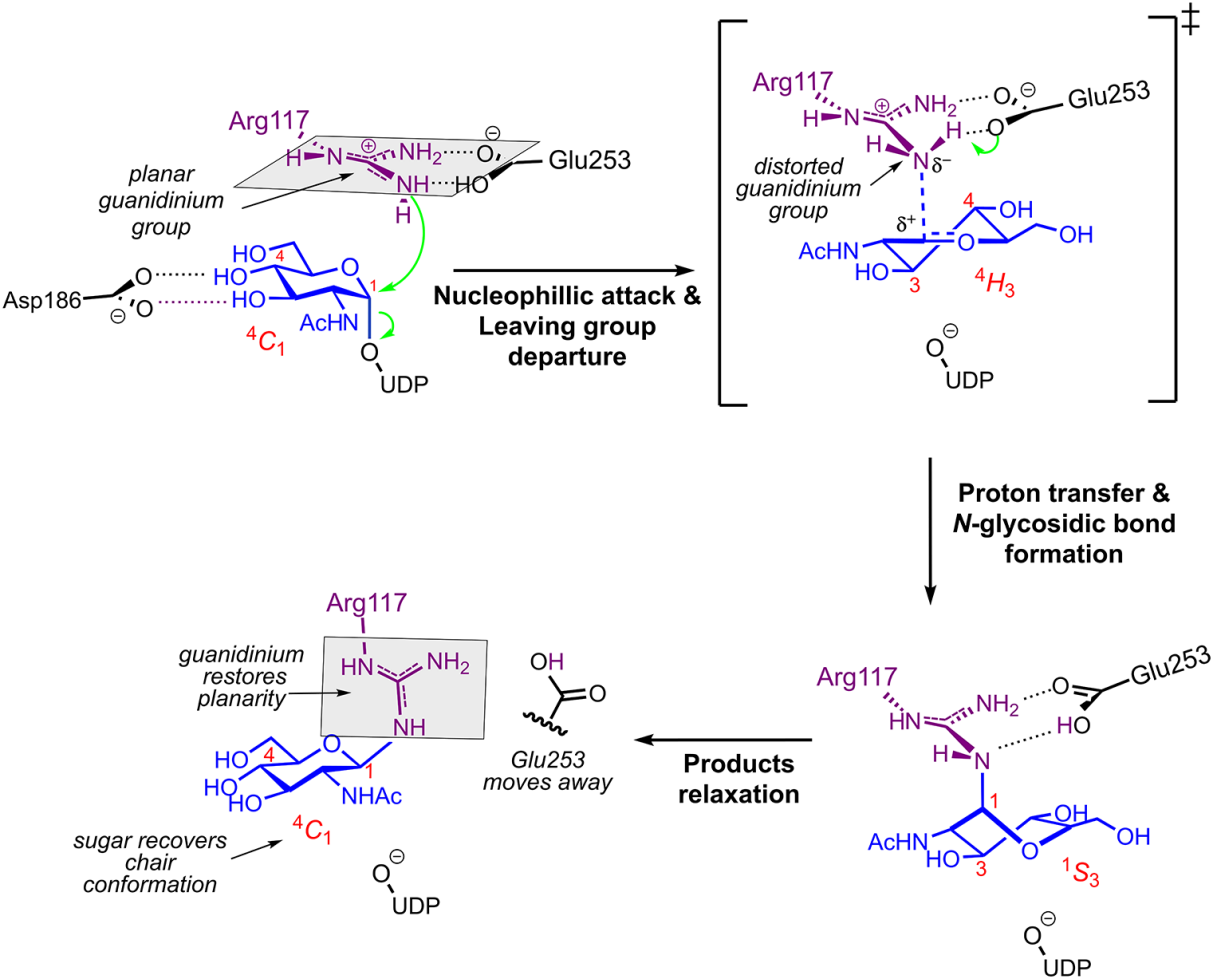
Reaction mechanism of arginine glycosylation catalyzed by NleB1 obtained in this work. The scheme illustrates the main electronic and structural rearrangements along the reaction coordinate, from the initial enzyme–substrate complex (top left) to products release (bottom left).

Contrary to our initial expectation of preattack deprotonation, the QM/MM metadynamics revealed a concerted but asynchronous inverting mechanism in which the C1–phosphate bond breaks first, followed by essentially simultaneous C1–N_η2_ bond formation and proton transfer after the transition state. We found no evidence of a lower energy route in which deprotonation precedes attack. The single-step free energy profiles, which show no stable oxocarbenium ion intermediate, also disfavor a stepwise S_N_1 mechanism. Other possibilities, such as substrate-assisted general-base catalysis via the β-phosphate, can be ruled out by the active-site geometry and post-TS proton transfer to Glu253. Under our simulation conditions, we also detect no signatures of retaining chemistry, despite reports of such behavior with suboptimal death-domain peptides in related systems.^16^

The critical nature of this Arg–Glu interaction is supported by site-directed mutagenesis experiments. Substitution of Glu253 with Asp reduces *k*_*cat*_ by 6.5-fold and increases *K*_m_ by 20-fold, resulting in a ∼ 130-fold drop in catalytic efficiency. MD simulations show that, although Asp253 can still act as a base, its shorter side chain weakens the bidentate interaction, fails to fully distort the guanidinium group, and often mispositions Arg117 for attack, while also delaying Asp253 detachment in the post-TS state. Together, these effects rationalize the reduced turnover and weaker substrate binding observed experimentally. In contrast, Asp186 contributes to sugar-donor binding rather than reactivity and therefore cannot compensate for the loss of Glu253. As a result, the delayed Asp253 detachment is not compensated, slowing product release and active site reset, thereby lowering catalytic efficiency. These results support that Glu253 is the catalytic base of the mechanism, and that the role of Asp186 is in binding the sugar moiety. However, it is possible to think that other death domains such as TRADD could have a slightly different active site configuration, in which Asp186 would be better placed to directly interact with the glycosylated Arg. We briefly explored this possibility by docking TRADD to NleB1 using HADDOCK and Rosetta, but we were unable to obtain a complex where the acceptor Arg was close enough to the GlcNAc to consider catalysis. Whether long time scale MD simulations or further modeling could achieve a suitable complex is beyond the scope of this work.

A mechanistic parallel can be drawn to a recent computational study on horseshoe crab arginine kinase (AK), which catalyzes phosphoryl (PO_3_^–^) transfer from ATP to free arginine.^54^ Although chemically distinct from glycosylation, the AK environment enables nucleophilic participation of arginine, with proton transfer from the guanidinium occurring only after the key bond-forming step. In NleB1, subsequent events—such as guanidinium planarization and GlcNAc conformational relaxation—also occur after product formation (Supplementary Figure 15). This suggests that late-stage rearrangements, beyond the chemical step itself, may be a common feature of enzymatic arginine activation. Recent findings in other enzymes further support this idea: for example, in the Zika virus helicase, ATP hydrolysis is followed by phosphate detachment that triggers a conformational shift essential for activity.^55^

## 5 Conclusions

Our simulations and experiments reveal that NleB1 catalyzes GlcNAc transfer to the death domain of FADD via a dissociative S_N_2 reaction mechanism in which Glu253 performs multiple coordinated functions along the catalytic cycle, including a role in product release. The glycosylation strategy adopted by NleB1 (distorting a poor nitrogen nucleophile in the ground state, catalyzing bond formation, and promoting product release) may be conserved in other arginine-modifying enzymes. Within the NleB/SseK family, the acidic residue equivalent to Glu253 is consistently Glu,^18^ and our Glu253Asp mutant confirms that Asp is suboptimal at this position (*k*_*cat*_↓, *K*_*m*_↑). In contrast, other arginine glycosyltransferasses such as EarP, an arginine rhamnosyltransferase, are expected to use an Asp as catalytic base.^56^ Here, a bidentate Asp–Arg contact^56,57^ could likewise serve to distort and align the guanidinium, enhancing its effective nucleophilicity and promoting an in-line S_N_2-type reaction. This, however, remains a hypothesis that will require targeted computational and experimental validation. Although EarP and NleB1 are structurally unrelated and use different donors and acceptors, such parallels may represent converged evolution among Arg-*N*-glycosyltransferases. More distantly, similar mechanistic principles may extend to protein arginine methyltransferases.^56,57^ Understanding this strategy provides a conceptual framework for designing mechanism-based inhibitors targeting the Arg–Glu interaction, with potential to attenuate bacterial pathogenicity.

## Supplementary Material







## Data Availability

Structure and trajectory files for all simulations can be accessed from DOI 10.5281/zenodo.17225948.

## Notes

Additional computational details; coordination-number definitions; average distances for Michaelis, TS, and product states; raw kinetic data and residuals; and supplementary MD and QM/MM figures and free-energy profiles (PDF)
Trajectory of the path-CV QM/MM metadynamics simulation of the Arg-*N*-glycosylation reaction (MP4)
Trajectory of the QM/MM metadynamics simulation of product relaxation (MP4)

## ⧧

Novo Nordisk Foundation Quantum Computing Programme (Niels Bohr Institute) and Department of Biology, University of Copenhagen, 2100 Copenhagen, Denmark (B.P.)
