# Supplementary material for: Mechanism of Bacterial Arginine N‑Glycosylation: A Chemically Challenging Post-Translational Modification
Source: ACS Catal. 2026 Jan 15;16(3):2550–62. doi: 10.1021/acscatal.5c07775 (PMC12888640; doi:10.1021/acscatal.5c07775)
Supplement: Supplementary file 1 [file cs5c07775_si_001.pdf]

SUPPORTING INFORMATION

Mechanism of bacterial arginine *N*-glycosylation: a chemically challenging post-translational modification

Beatriz Piniello[1,‡], Ana García-García[2], Fabio Pietrucci[3], Ramón Hurtado-Guerrero[2,*], Carme Rovira[1,4,*]

[1]Departament de Química Inorgànica i Orgànica (Secció de Química Orgànica) and Institut de Química Teòrica i Computacional (IQTCUB), Universitat de Barcelona, Martí i Franquès 1, 08028 Barcelona, Spain. [2]Institute of Biocomputation and Physics of Complex Systems (BIFI), University of Zaragoza, Mariano Esquillor s/n, Campus Rio Ebro, Edificio I+D, 50018 Zaragoza, Spain; Fundación ARAID, Zaragoza, 50018, Spain; Copenhagen Center for Glycomics, Department of Cellular and Molecular Medicine, University of Copenhagen, DK-2200 Copenhagen, Denmark. [3]Sorbonne Université, Muséum National d'Histoire Naturelle, UMR CNRS 7590, Institut de Minéralogie, de Physique des Matériaux et de Cosmochimie, IMPMC, 75005 Paris, France. [4]Institució Catalana de Recerca i Estudis Avançats, Passeig Lluís Companys 23, 08010 Barcelona, Spain.

Keywords: Enzymes • N-glycosylation • Carbohydrates • Glycosyltransferases • Quantum mechanics/Molecular mechanics • metadynamics

SUPPLEMENTARY TABLES

Supplementary Table 1. Coordination numbers for each pair of atoms (column-row) for the two nodes (Reactants, R, and Products, P) used in the initial path CV metadynamics.

A

**Node 1** (MC)	C1	$O_P/O_P'/O_P''$	$N_{\eta 2}$	$H_{\eta 2}$	$O1_{Glu253}/O2_{Glu253}$
C1	0	0.9	0.01	0	0
$O_P/O_{P'}/O_{P''}$	0.9	0.85	0	0	0
$N_{\eta 2}$	0.01	0	0	1.6	0.13
$H_{\eta 2}$	0	0	1.6	0.25	0.18
$O1_{Glu253}/O2_{Glu253}$	0	0	0.13	0.18	0.52

B

**Node 2** (P)	C1	$O_P/O_P'/O_P''$	$N_{\eta 2}$	$H_{\eta 2}$	$O1_{Glu253}/O2_{Glu253}$
C1	0	0	0.8	0.07	0
$O_P/O_{P'}/O_{P''}$	0	0.85	0	0	0
$N_{\eta 2}$	0.8	0	0	0.9	0.1
$H_{\eta 2}$	0.07	0	0.9	0.06	0.85
$O1_{Glu253}/O2_{Glu253}$	0	0	0.1	0.85	0.52

Supplementary Table 2. Average distances of MC and P around the corresponding free energy minimum (±0.05 around the CV value). In the case of the TS, values correspond to those obtained from committor analysis.

	MC	TS	P
C1-O_P	1.47 ± 0.06	3.40	3.38 ± 0.25
C1-$N_{\eta 2}$	3.89 ± 0.23	2.04	1.52 ± 0.04
$N_{\eta 2}$-$H2_{\eta 2}$	1.05 ± 0.05	1.13	1.98 ± 0.13
$O1_{Glu253}$-$H2_{\eta 2}$	1.76 ± 0.21	1.46	1.00 ± 0.02
$N_{\eta 1}$-$H2_{\eta 1}$	1.06 ± 0.04	1.09	1.03 ± 0.03
$O2_{Glu253}$-$H2_{\eta 1}$	1.77 ± 0.18	1.54	2.14 ± 0.36
C1-O5	1.41 ± 0.04	1.34	1.41 ± 0.03

Supplementary Table 3. Raw experimental velocity and substrate concentration data, along with corresponding residuals from kinetic model fitting. Predicted rates were calculated with the substrate-inhibition model:

$$v_{pred} = \frac{V_{\max}[S]}{K_m + [S](1 + \frac{[S]}{K_i})}$$

Residuals were defined as:

$$Residual = v_{obs} - v_{pred}$$

for each replicate at each substrate concentration.

A) Data for **NleB1 WT**:

**Concentration (µM)**	**Observed 1 (Specific activity) umol/min*mg**	**Residual 1**	**Observed 2 (Specific activity) umol/min*mg**	**Residual 2**	**Predicted**
0	0.000	0.000	0.000	0.000	0.000
5	1.209	0.155	0.821	-0.233	1.054
10	1.222	-0.407	1.573	-0.056	1.630
25	2.783	0.372	2.742	0.331	2.411
100	3.067	0.035	2.815	-0.217	3.033
200	2.873	-0.120	3.018	0.026	2.992
400	2.444	-0.271	2.699	-0.016	2.715
600	2.429	-0.022	2.583	0.133	2.450
800	2.546	0.320	2.404	0.178	2.226
1000	1.894	-0.142	1.867	-0.170	2.037

Residual 1: Difference between Observed 1 and model-predicted value at each substrate concentration.
Residual 2: Difference between Observed 2 and model-predicted value at each substrate concentration.

B) Data for **E253D NleB1:**

**Concentration (μM)**	**Observed 1 (Specific activity) umol/min*mg**	**Residual 1**	**Observed 2 (Specific activity) umol/min*mg**	**Residual 2**	**Predicted**
0	0.000	0.000	0.000	0.000	0.000
25	0.025	-0.026	0.024	-0.027	0.051
50	0.076	-0.016	0.075	-0.017	0.092
100	0.111	-0.040	0.077	-0.074	0.151
200	0.159	-0.049	0.124	-0.084	0.209
400	0.212	-0.013	0.174	-0.051	0.224
600	0.257	0.052	0.241	0.035	0.206
800	0.193	0.009	0.149	-0.035	0.184

SUPPLEMENTARY FIGURES

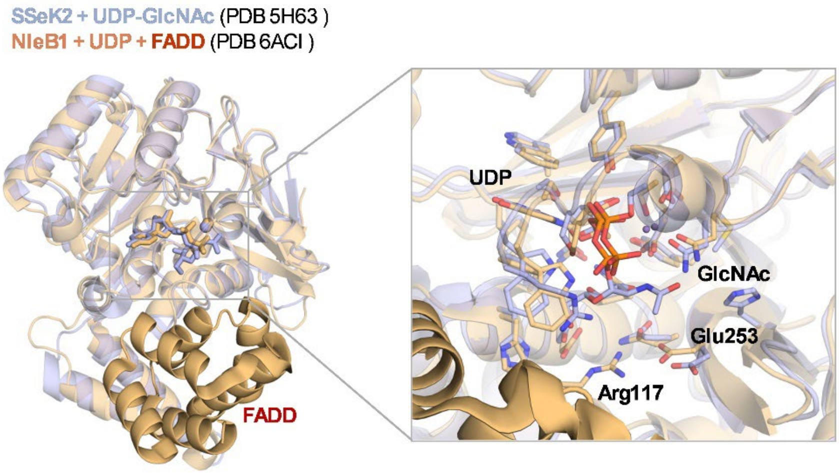

Supplementary Figure 1. Superposition of the two crystal structures (5H63 and 6ACI),[1, 2] used to build the Michaelis complex.

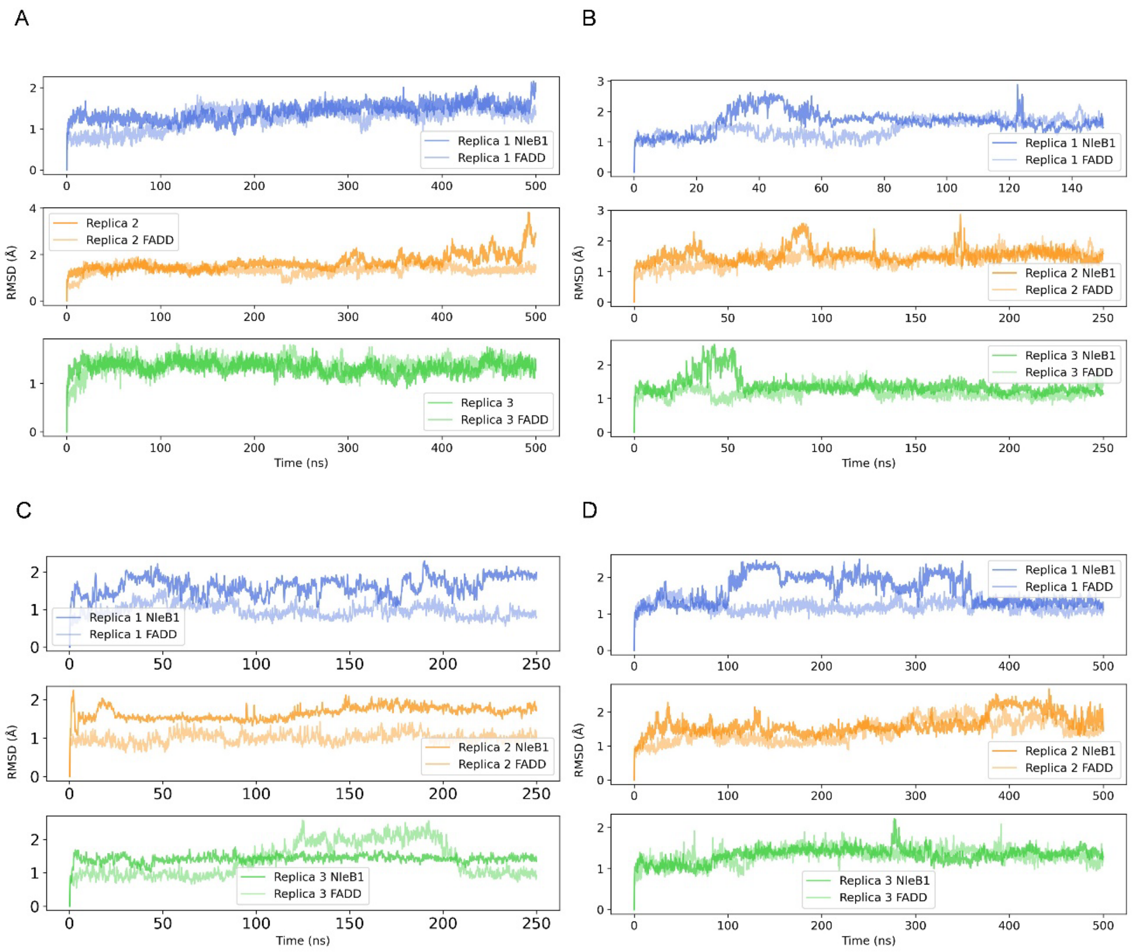

Supplementary Figure 2. RMSD evolution during the classical MD simulations of NleB1 complexes. (A, B) Michaelis complex (*cis* and *trans* conformations of *N*-acetylglucosamine (NHAc) were considered, respectively). (C) Product complex (*trans* NHAc). (D) Glu253Asp mutant (*trans* NHAc).

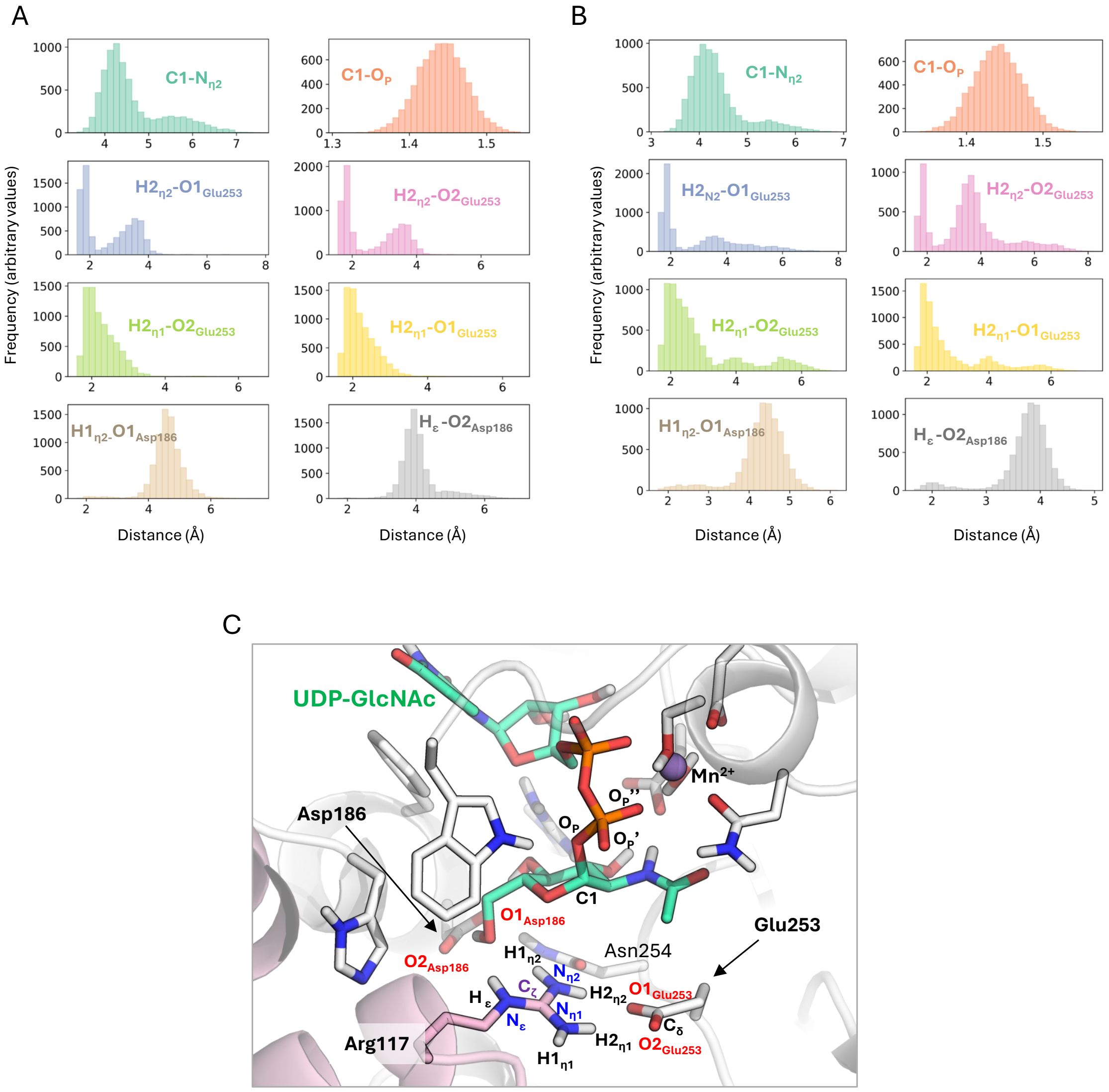

Supplementary Figure 3. (A) Evolution of relevant catalytic distances during classical MD simulations of the Michaelis complex (*cis* NHAc). (B) Same for *trans* NHAc. (C) Atom labeling.

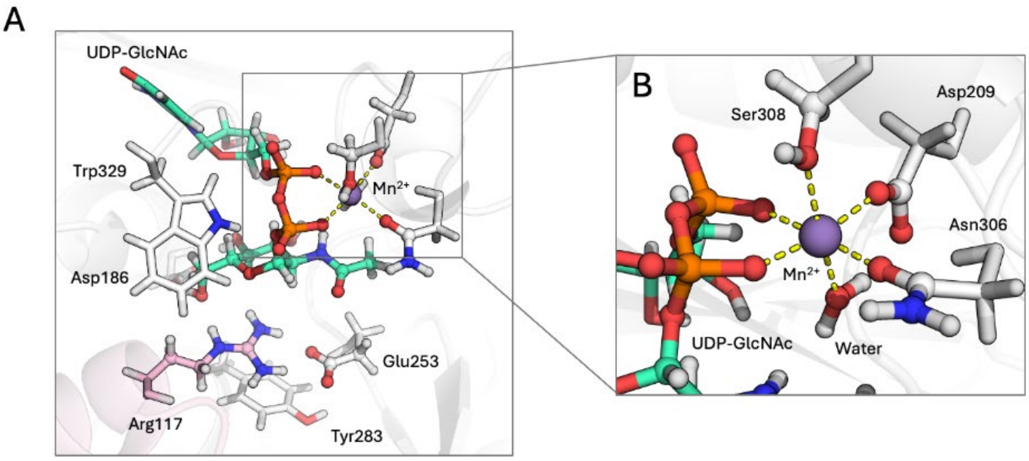

Supplementary Figure 4. (A) Atoms included in the QM region (in sphere representation). (B) Detail of Mn^{2+} coordination sphere.

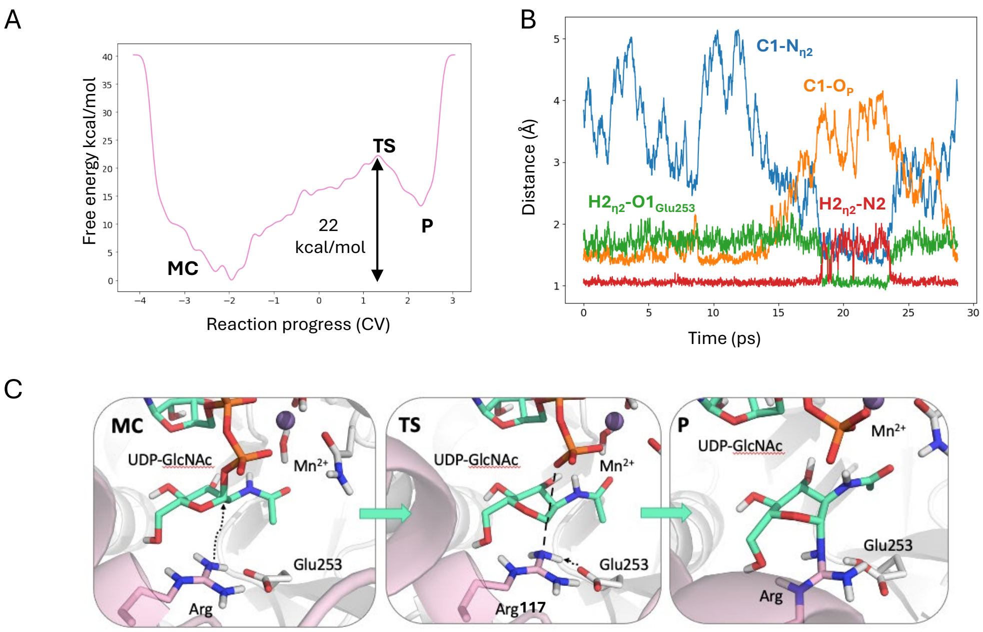

Supplementary Figure 5. (A) Free energy profile of the 1-CV QM/MM metadynamics simulation of the glycosylation reaction. (B) Evolution of relevant catalytic distances along the simulation. (C) Snapshots of the active site for the most relevant states.

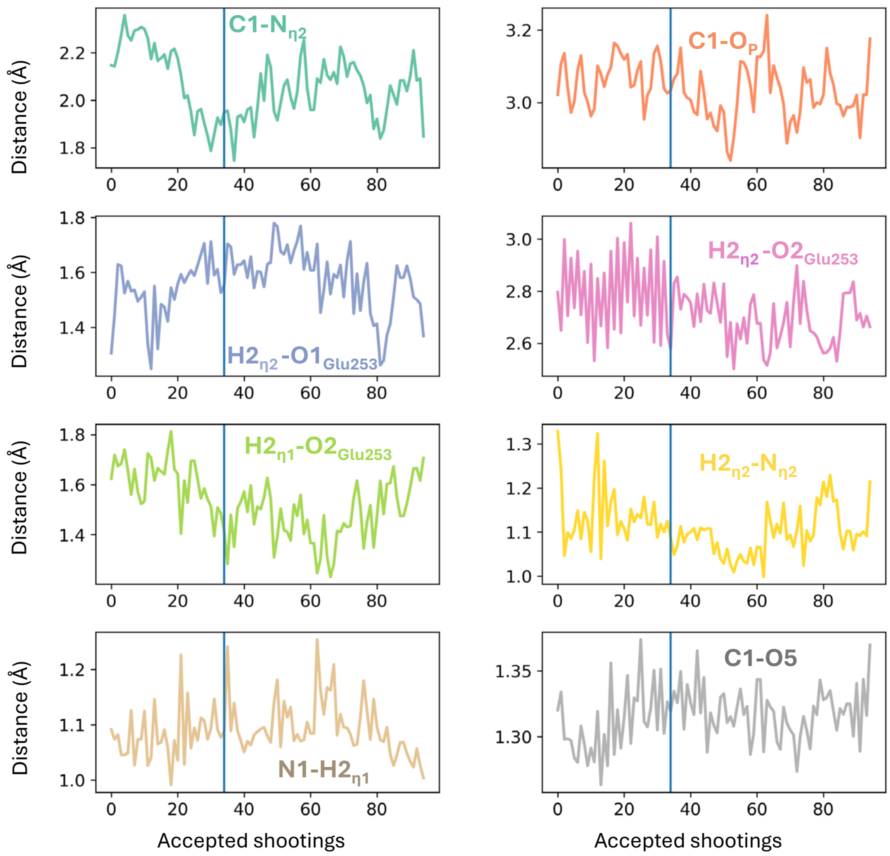

Supplementary Figure 5. Evolution of distances during the accepted shootings, in the aimless shooting simulations used to train the path CV. The blue line indicates the change in the time step for the aimless shooting algorithm (see Computational details).

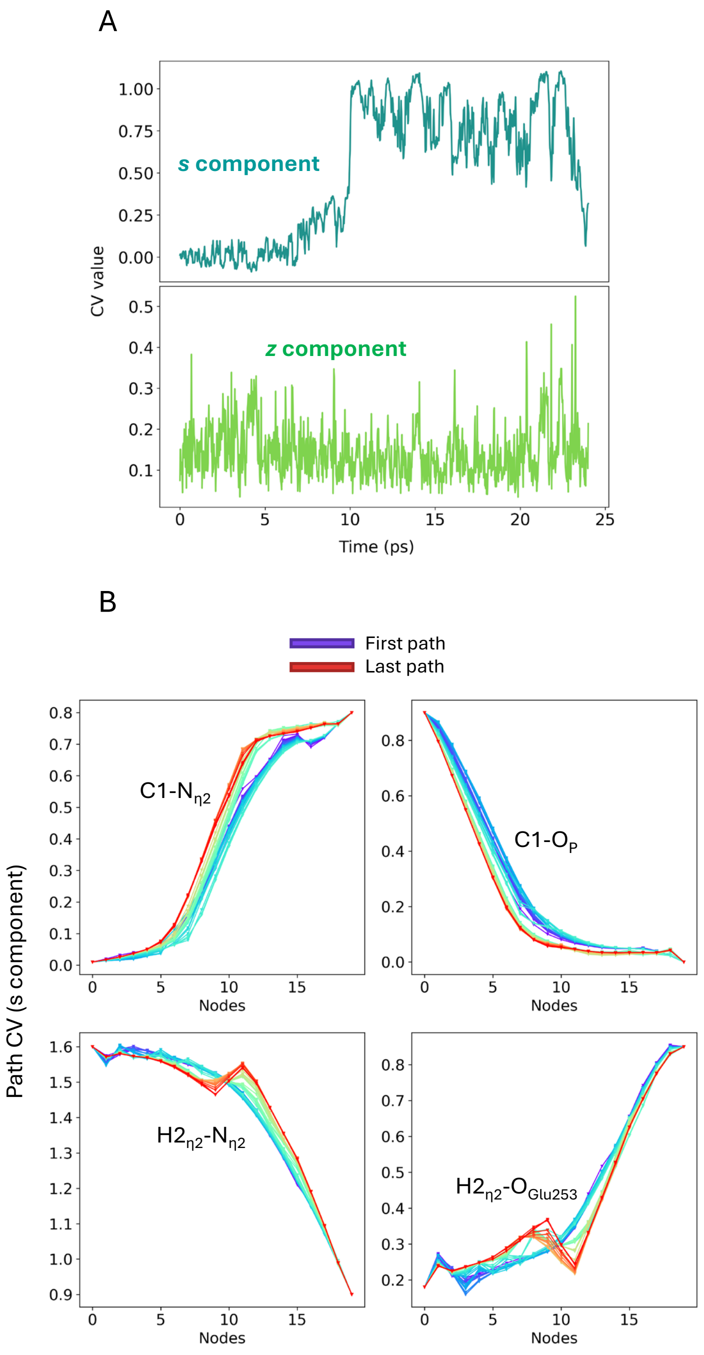

Supplementary Figure 7. (A) Evolution of the two components of the path CV (being s the biased variable) during the QM/MM path metadynamics simulation. (B) Evolution of the path as it is updated during the metadynamics simulation, considering the nucleophilic attack and glycosidic sugar-phosphate bond cleavage (top) and proton transfer (bottom). Each colored bead represents a node, and the evolution of the path in time is represented by different colors. The initial path is represented in violet color, whereas the last converged path is in red.

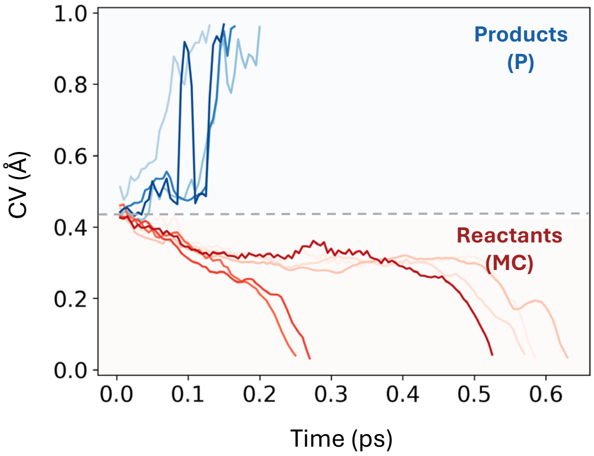

Supplementary Figure 8. Committor analysis results of the TS candidate from the path CV QM/MM metadynamics simulation.

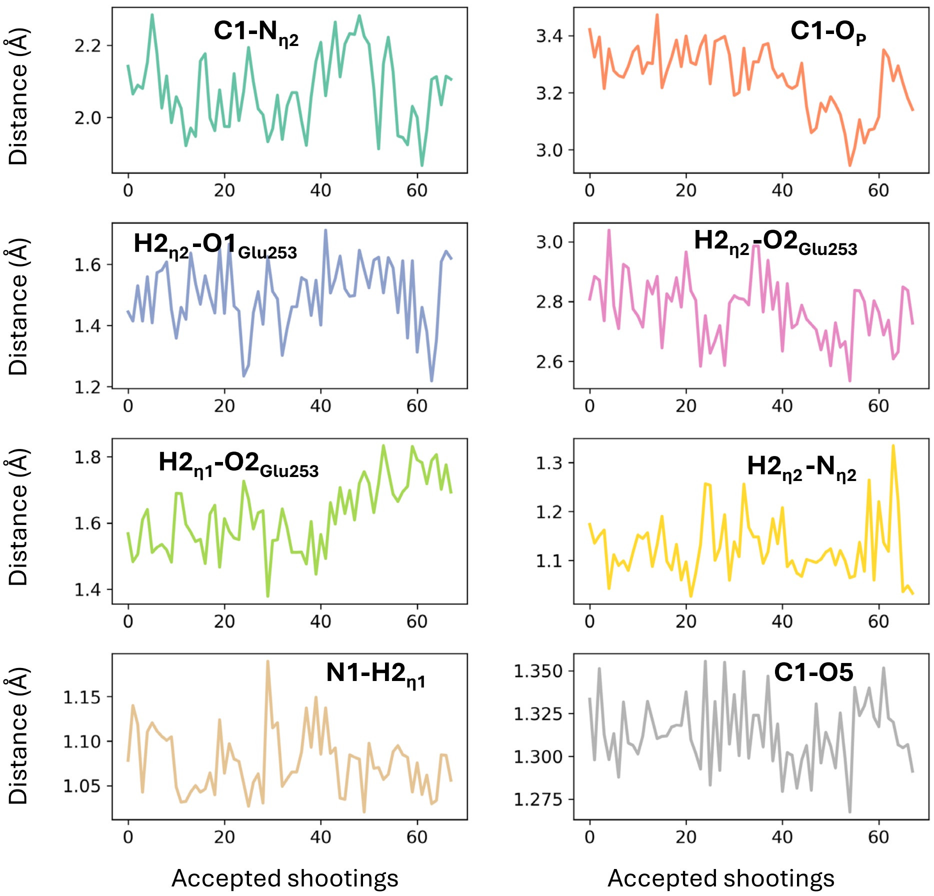

Supplementary Figure 9. Accepted configurations of the aimless shooting simulations starting from the TS of the path CV QM/MM metadynamics of the glycosylation reaction. A total of 122 simulations were launched, with an acceptance ratio of 57%.

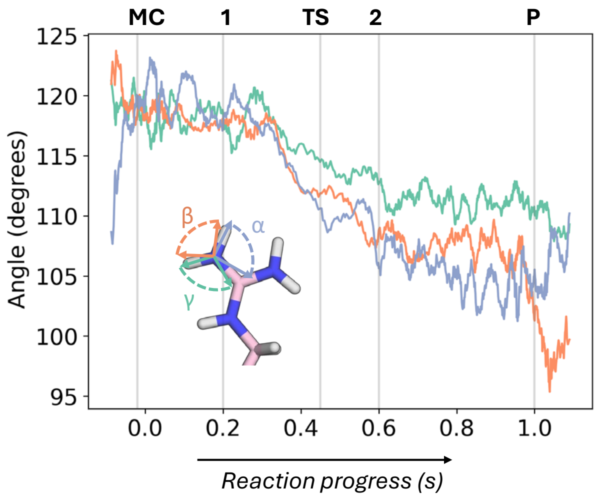

Supplementary Figure 10. Evolution of the three angles involving the arginine $N_{\eta2}$ atom: <C_{ζ}-$N_{\eta2}$-$H2_{\eta2}$ (blue), <C_{ζ}-$N_{\eta2}$-$H1_{\eta2}$ (green) and <$H1_{\eta2}$-$N_{\eta2}$-$H2_{\eta2}$ (orange) along the reaction coordinate. See atom labels in Supporting Figure 3. Results obtained from the 20-node QM/MM path metadynamics simulation.

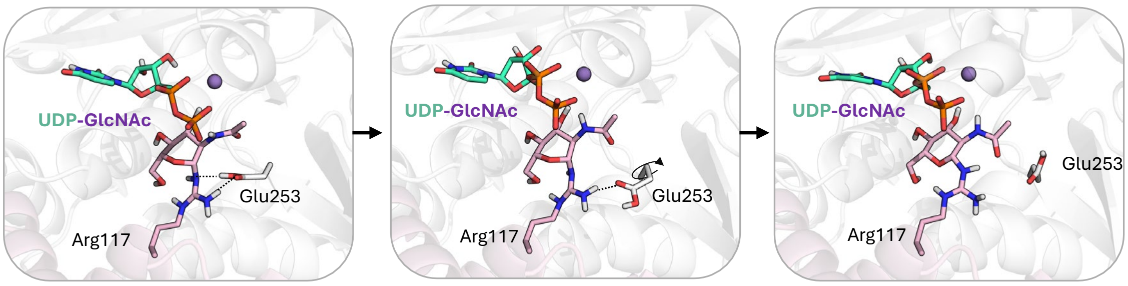

Supplementary Figure 11. Snapshots from the unbiassed classical MD of the products obtained. (A) Initial state, featuring the double hydrogen bond between Glu253 and Arg117. (B) The hydrogen bond weakens. (C) Glu253 moves away from Arg117 and, simultaneously, the sugar ring adopts a chair conformation and the guanidinium group recovers the planar conformation.

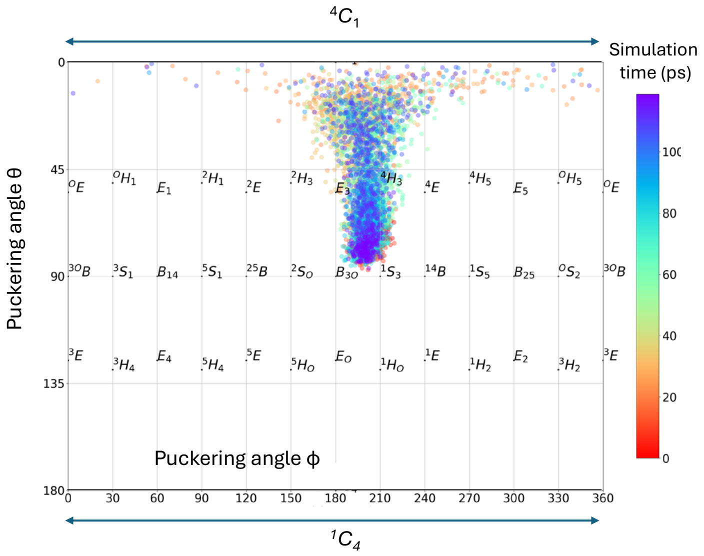

Supplementary Figure 12. Conformations visited by the sugar ring during the QM/MM metadynamics simulation of product relaxation, revealing a $^{1}S_{3}/B_{3,O} \longrightarrow {}^{4}H_{3} \longrightarrow {}^{4}C_{1}$ conformational itinerary.

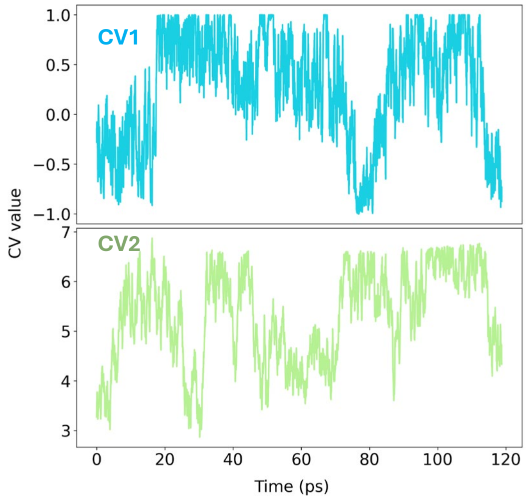

Supplementary Figure 13. Evolution of CVs in the QM/MM metadynamics simulation of product relaxation. CV1 = cos Ω; CV2 = $N_{\eta2}$- C_{δ} distance, in Å.

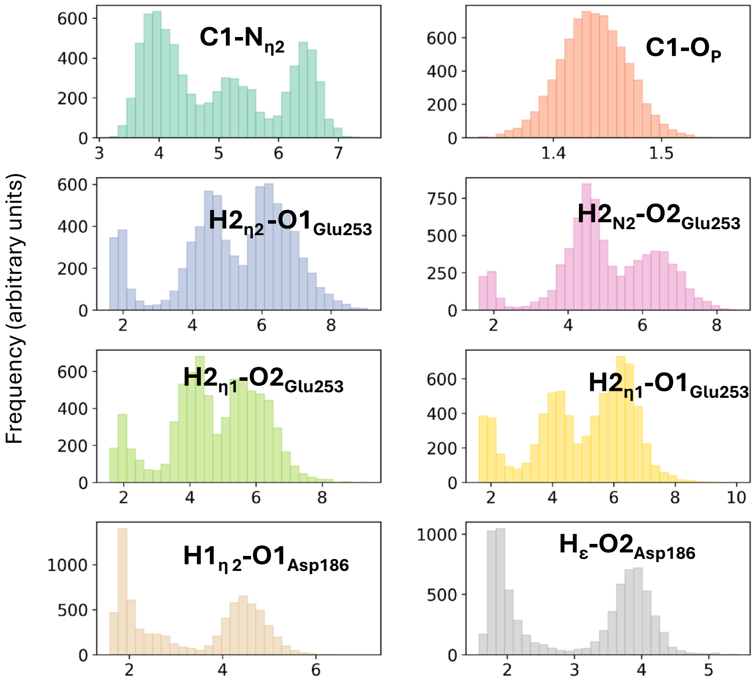

Supplementary Figure 14. Distribution of relevant catalytic distances obtained in the classical MD simulation of NleB1 Glu253Asp.

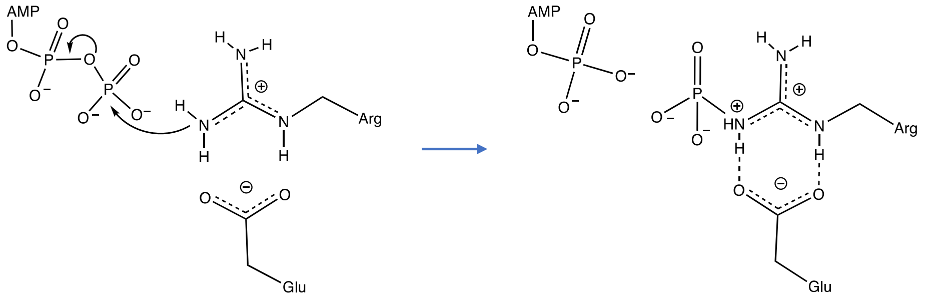

Supplementary Figure 15. Mechanism proposed for arginine kinase (KS) by Falcioni and co-workers.[3]

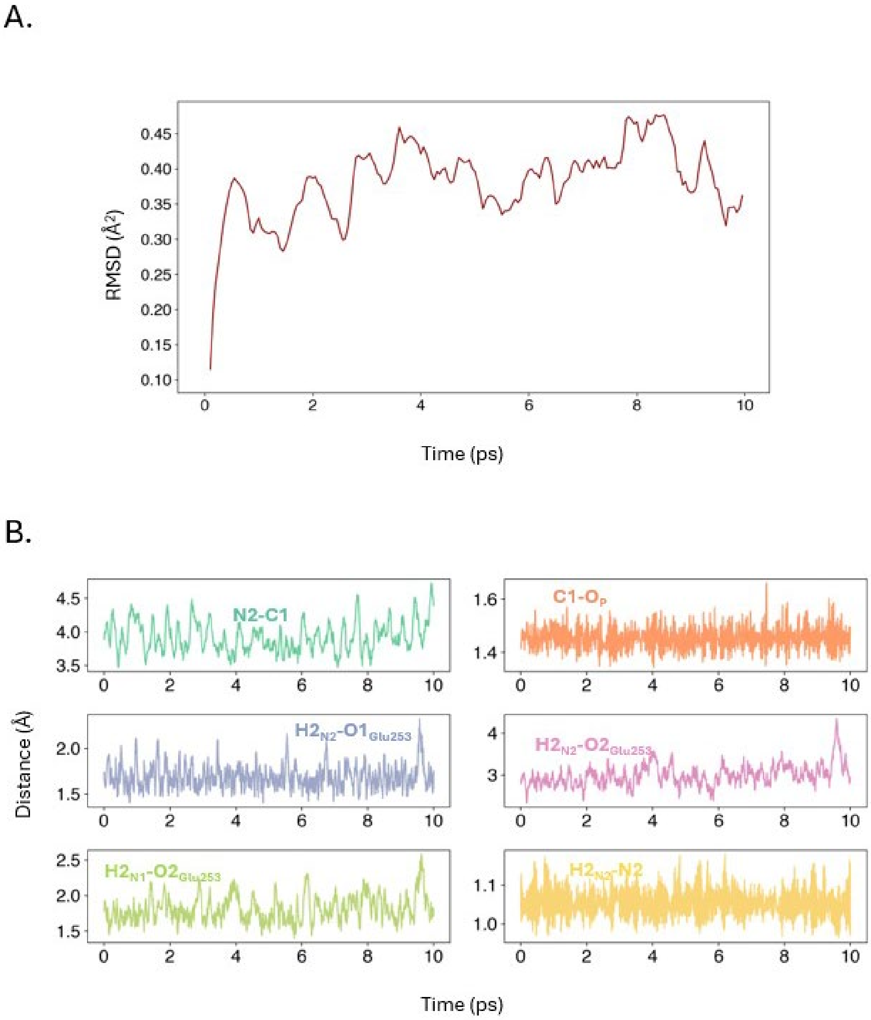

Supplementary Figure 16. (A) RMSD of QM atoms during the QM/MM equilibration. (B) Relevant active site distances evolution during QM/MM equilibration.

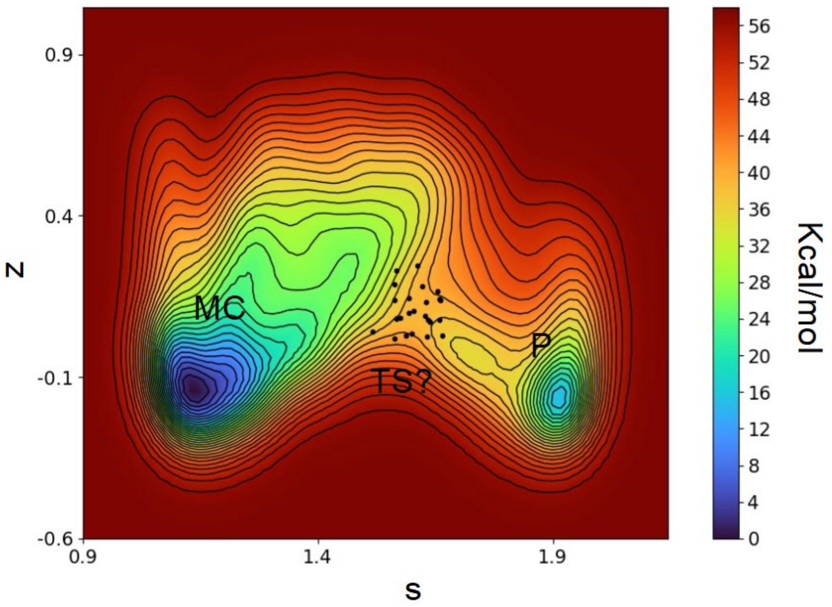

Supplementary Figure 17. Metadynamics bias obtained from the first path CV metadynamics simulation. Black dots are TS candidates screened for starting the aimless shooting simulations.

SUPPLEMENTARY VIDEOS

Supplementary video 1. Trajectory of the path CV QM/MM metadynamics simulation of the reaction mechanism.

Supplementary video 2. Trajectory of the QM/MM metadynamics simulation of product relaxation.

References

(1) Ding, J.; Pan, X.; Du, L.; Yao, Q.; Xue, J.; Yao, H.; Wang, D.-C.; Li, S.; Shao, F. Structural and Functional Insights into Host Death Domains Inactivation by the Bacterial Arginine GlcNAcyltransferase Effector. *Mol. Cell* **2019**, *74*, 922-935.e926.
(2) Park, J. B.; Kim, Y. H.; Yoo, Y.; Kim, J.; Jun, S.-H.; Cho, J. W.; El Qaidi, S.; Walpole, S.; Monaco, S.; García-García, A. A.; Wu, M.; Hays, M. P.; Hurtado-Guerrero, R.; Angulo, J.; Hardwidge, P. R.; Shin, J.-S.; Cho, H.-S. Structural basis for arginine glycosylation of host substrates by bacterial effector proteins. *Nat. Commun.* **2018**, *9*, 4283.
(3) Falcioni, F.; Molt, R. W., Jr.; Jin, Y.; Waltho, J. P.; Hay, S.; Richards, N. G. J.; Blackburn, G. M. Arginine Kinase Activates Arginine for Phosphorylation by Pyramidalization and Polarization. *ACS Catal.* **2024**, *14*, 6650-6658.
